# The Limbic System in Children and Adolescents With Attention-Deficit/Hyperactivity Disorder: A Longitudinal Structural Magnetic Resonance Imaging Analysis

**DOI:** 10.1016/j.bpsgos.2023.10.005

**Published:** 2023-11-02

**Authors:** Michael Connaughton, Erik O’Hanlon, Timothy J. Silk, Julia Paterson, Aisling O’Neill, Vicki Anderson, Robert Whelan, Jane McGrath

**Affiliations:** a Department of Psychiatry, School of Medicine, Trinity College Dublin, Dublin, Ireland; b Trinity College Institute of Neuroscience, Trinity College Dublin, Dublin, Ireland; c Department of Psychiatry, Royal College of Surgeons in Ireland, Dublin, Ireland; d Department of Developmental Neuroimaging, Murdoch Children’s Research Institute, Melbourne, Victoria, Australia; e Centre for Social and Early Emotional Development and School of Psychology, Deakin University, Geelong, Victoria, Australia; f Department of Psychology, Royal Children’s Hospital, Melbourne, Victoria, Australia; g Global Brain Health Institute, Trinity College Dublin, Dublin, Ireland

**Keywords:** Attention-deficit/hyperactivity disorder, Brain development, Emotion dysregulation, Limbic system, Magnetic resonance imaging, Structural neuroimaging

## Abstract

**Background:**

During childhood and adolescence, attention-deficit/hyperactivity disorder (ADHD) is associated with changes in symptoms and brain structures, but the link between brain structure and function remains unclear. The limbic system, often termed the “emotional network,” plays an important role in a number of neurodevelopmental disorders, yet this brain network remains largely unexplored in ADHD. Investigating the developmental trajectories of key limbic system structures during childhood and adolescence will provide novel insights into the neurobiological underpinnings of ADHD.

**Methods:**

Structural magnetic resonance imaging data (380 scans), emotional regulation (Affective Reactivity Index), and ADHD symptom severity (Conners 3 ADHD Index) were measured at up to 3 time points between 9 and 14 years of age in a sample of children and adolescents with ADHD (*n* = 57) and control children (*n* = 109).

**Results:**

Compared with the control group, the ADHD group had lower volume of the amygdala (left: β standardized [β_std] = −0.38; right: β_std = −0.34), hippocampus (left: β_std = −0.44; right: β_std = −0.34), cingulate gyrus (left: β_std = −0.42; right: β_std = −0.32), and orbitofrontal cortex (right: β_std = −0.33) across development (9–14 years). There were no significant group-by-age interactions in any of the limbic system structures. Exploratory analysis found a significant Conners 3 ADHD Index-by-age interaction effect on the volume of the left mammillary body (β_std = 0.17) in the ADHD group across the 3 study time points.

**Conclusions:**

Children and adolescents with ADHD displayed lower volume and atypical development in limbic system structures. Furthermore, atypical limbic system development was associated with increased symptom severity, highlighting a potential neurobiological correlate of ADHD severity.

Attention-deficit/hyperactivity disorder (ADHD) is a common neurodevelopmental disorder with global prevalence estimates of 5% to 7% in children and adolescents (1). Alongside the core behavioral symptoms of inattention, hyperactivity, and impulsivity, ADHD is also associated with emotional dysregulation (2,3). Emotional dysregulation is an individual’s inability to adjust their emotional state in a manner that promotes adaptive, goal-oriented behaviors (3). In individuals with ADHD, emotional dysregulation during childhood is associated with increased rates of anxiety, mood disorders, disruptive behavior disorders, and drug abuse in adulthood (4). During the transition from childhood to adolescence, ADHD symptoms undergo notable change, with hyperactive and impulsive symptoms often declining (5,6) and inattentive symptoms tending to persist (7). While the trajectories of emotional dysregulation during this period remain unclear, studies suggest that because the core symptoms of ADHD improve into adulthood, emotional dysregulation may show parallel improvements (4).

In parallel with these symptom changes, it is known that the human brain undergoes significant structural changes in gray matter volume, surface area, and cortical thickness (8, 9, 10) during the transition from childhood to adolescence. Interestingly, longitudinal neuroimaging research in individuals with ADHD has consistently revealed deviations in brain development. Compared with control participants, individuals with ADHD exhibit reduced or delayed cortical maturation in areas associated with attention, impulse control, and executive functioning (11, 12, 13, 14). Two leading neurodevelopmental theories of ADHD include the maturation delay hypothesis (11, 12, 13) and the convergence model (14). The maturation delay hypothesis posits that relative to control participants, individuals with ADHD show an approximately 3-year delay in cortical development, particularly in the frontal, temporal, and parietal lobes (11), with this delay being most pronounced during childhood (11,12). The convergence model suggests that symptom improvement or remission in ADHD is associated with the normalization of neural features that were atypical in early childhood (14). This model also integrates the concept of delayed maturation in ADHD, suggesting that it is underpinned by a correction of earlier developmental lags (14). The critical difference between the 2 models is that according to the convergence model, children with persistent ADHD show ongoing nonprogressive differences in brain structures across the lifetime compared with control participants (14).

Although these developmental models provide insights into the neurobiological underpinnings of ADHD symptom progression, most longitudinal studies have focused on cortical structures (11,12). As a result, our understanding of these models in relation to subcortical structures and networks remains largely incomplete (15,16). This gap is particularly surprising considering that functional imaging research suggests that abnormalities in subcortical brain regions may be present from the onset of ADHD and continue throughout life irrespective of symptom change (17). To further understand the neurobiological mechanisms of ADHD, it is essential to extend these neurodevelopmental models to subcortical structures and networks.

The limbic system is a group of interconnected cortical (cingulate gyrus and orbitofrontal cortex) and subcortical (amygdala, hippocampus, mammillary bodies, and anterior thalamic nuclei) structures (see network schematic in Figure 1) involved in the processes of emotion and cognition (18,19). While atypical limbic system structure and function have been identified in various neurodevelopmental disorders (20), their specific roles and influence within the context of ADHD remains largely unknown. This lack of investigation is surprising given the high prevalence of emotion dysregulation observed in individuals with ADHD (3). Additionally, some recent studies have found that core symptoms of ADHD, such as impulsivity and attention deficits, are associated with structural and functional abnormalities within limbic system regions (21, 22, 23). Therefore, understanding the developmental trajectories of this prominent brain network could shed light on the underlying neural mechanisms responsible for both the core symptoms and associated emotional dysregulation, thus offering insights into the disorder’s pathogenesis.

**Figure 1:**
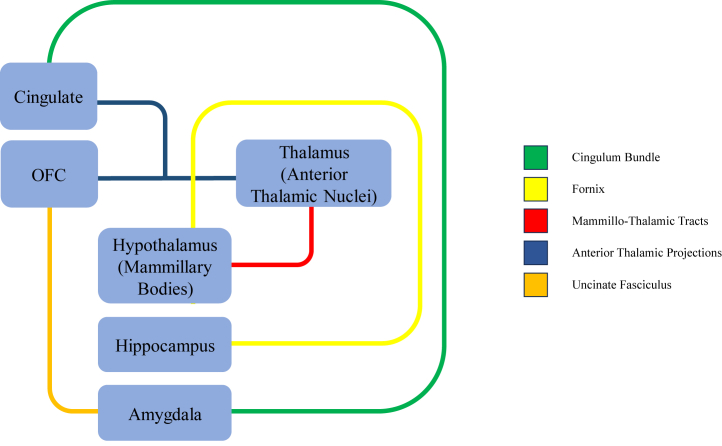
Schematic of the limbic system. Graphical illustration of the limbic system alongside its primary pathways. The colors of the pathways match the tracts specified in the legend. OFC, orbitofrontal cortex.

This study aims to provide novel insights into the structural development of this crucial brain network and also further our understanding of the relationship between ADHD symptoms and brain structure during the pivotal transition from childhood into adolescence. Using longitudinal structural magnetic resonance imaging (MRI) data, acquired across 3 time points, this study investigates the volumetric development of limbic system structures in children with ADHD and control participants. The hypothesis was that compared with control participants, individuals with ADHD would display lower volumes and delayed development in limbic system structures during the transition from childhood to adolescence. This study also aims to investigate brain-behavior relationships through a series of exploratory analyses to better understand the functional impact of the limbic system in ADHD.

## Methods and Materials

### Sample

The data in this study were collected as part of the Neuroimaging of the Children’s Attention Project (NICAP) (24). NICAP is a longitudinal study in which children with and without ADHD were assessed at 3 time points, at approximately 18-month intervals, from ages 9 to 14 years. The study protocol involves a multimodal MRI scan and a battery of questionnaires across various functional domains at each study time point. This study focused on individuals with a persistent ADHD diagnosis across the study time points. As such, the participants included in the ADHD group received a confirmed clinical ADHD diagnosis based on a clinically administered National Institute of Mental Health Diagnostic Interview Schedule for Children (25) at each assessment (recruitment [3 years prior to imaging], wave 1, and wave 3 imaging time points). Participants in the control group did not meet the diagnostic criteria for ADHD at any study time point. Written informed consent was obtained from participants’ parents/guardians before enrollment. Ethical approval was obtained from the Royal Children’s Hospital Melbourne Human Research Ethics Committee (HREC #34071).

### Image Acquisition

A 30-minute mock scan session was completed before scanning to familiarize each participant with the MRI environment and procedures. All neuroimaging data were collected at a single site at the Murdoch Children’s Research Institute at the Royal Children’s Hospital, Melbourne, on a 3T Siemens scanner using a 32-channel head coil; however, while the first 2 waves were collected on a TIM Trio scanner, the third wave was collected after an upgrade to a MAGNETOM Prisma scanner [previous papers on this cohort have reported minimal effects of scanner upgrade (26)]. T1-weighted volumes were collected using a multi-echo magnetization-prepared rapid acquisition gradient-echo sequence with in-scanner motion correction (repetition time = 2530 ms, echo time = 1.77, 3.51, 5.32, 7.2 ms, matrix = 256 × 232, slices = 176, voxel size = 0.9 mm^3^, flip angle = 7°). T2-weighted volumes were obtained using a T2 SPACE (sampling perfection with application-optimized contrast with flip angle evolution) protocol (repetition time = 3200 ms, echo time = 532, matrix = 256 × 230, slices = 176, voxel size = 0.9 mm^3^). T1- and T2-weighted volumes were used to provide optimal sensitivity and increase the accuracy of subcortical brain reconstruction (27,28). In cases where head motion during the scan was high, multiple scans were acquired until a suitable scan was completed.

### Image Processing

All T1 and T2 structural MRI scans were manually inspected and rated for artifacts, data quality issues, and excessive head motion (see the Supplement). FreeSurfer software (http://surfer.nmr.mgh.harvard.edu/) was used to isolate the structures of the limbic system. FreeSurfer analyses were performed using a RedHat-based scientific Linux 7 on the high-performance computing system at Trinity College Dublin, Ireland. All MR images were processed using FreeSurfer’s recon -all function (version 7.2) for full cortical reconstruction and brain segmentation (29,30). The Desikan-Killiany-Tourville atlas was used for brain parcellations (31). Additional FreeSurfer segmentation tools were used to extract the anterior thalamic nuclei (32) and mammillary bodies (33). The following bilateral limbic system structures were isolated: amygdala, hippocampus, mammillary bodies, anterior thalamic nuclei, cingulate gyrus (sum of cingulate gyrus parcellations), and orbitofrontal cortex (sum of orbitofrontal cortex parcellations). Before statistical analyses, all data points were Winsorized (34,35) (i.e., set to ±3 standard deviations above or below the mean) to minimize the effects of extreme outliers.

### Behavioral Measures

The parent-reported Conners 3 ADHD Index (CAI) (36) was completed at all 3 time points. The CAI is a 10-item questionnaire that measures the presence of core ADHD symptoms, with a higher score reflecting a greater presence of symptoms. The Affective Reactivity Index (ARI) (37), a 6-item questionnaire, was completed at study time points 1 and 2. The ARI measures emotional dysregulation and irritability (37), with a higher ARI score indicating greater emotional dysregulation (37).

### Statistical Analysis

Primary statistical analyses were performed using the R software package (version 4.1.1). To measure between-group differences in volume of limbic system structures, linear mixed-effects modeling (LME) was performed using the lme4 package in R (version 1.1-27.1) (38). LME can accommodate unbalanced data (39,40), which is a common issue in longitudinally designed studies. An essential procedure of LME is data-driven model selection, in which the goal is to identify a parsimonious model (i.e., high goodness of fit using as few explanatory variables as possible) to reduce the risk of a type 1 error (41). An established top-down LME model selection procedure was used to select the optimal model for each structure of the limbic system (41,42). Details of the model selection procedure and tested models are provided in the Supplement (Table S1). In all the optimal models, a robust 2-stage false discovery rate (FDR) (43) procedure was employed to correct for multiple comparisons at *q* = .05. Two-stage FDR correction was conducted using the MuToss package (44) in R (version 4.1.1). Sensitivity analyses were performed to assess the potential impact of confounding factors (case-control sex imbalance and ADHD medication status) on the primary statistical analyses (see Supplement).

Exploratory analyses were performed to examine the relationship between limbic system volumes and ADHD symptoms (CAI and ARI) in children and adolescents with ADHD. These relationships were investigated using LME via the lme4 package in R (version 1.1-27.1) (38). The chosen LME model, illustrated as model FX3b in Table S1, evaluated whether changes in limbic system volumes varied over time based on ADHD symptom severity, incorporating an age-by-ADHD symptoms interaction term. This model also adjusted for covariates of age, sex, and intracranial volume. To mitigate the effects of multiple comparisons, a 2-stage FDR correction was applied using the MuToss package (44) in R (version 4.1.1).

## Results

### Study Sample

The final data consisted of 380 scans from 166 individuals (children with ADHD *n* = 57, control children *n* = 109) scanned across 3 time points between the ages of 9 and 14 years (Table 1).

**Table 1:** Study Cohort Characteristics

Variable	ADHD	Control	Test of Significance
Total Scans	123	257	–
Scans Wave 1	50	99	–
Scans Wave 2	48	94	–
Scans Wave 3	25	64	–
Medication Use at Any Wave, %	43%	0%	–
Female Wave 1, %	20%	43%	*p* = .005
Female Wave 2, %	22%	43%	*p* = .021
Female Wave 3, %	33%	42%	*p* = .382
Age Wave 1, Years, Mean	10.35	10.38	*p* = .681
Age Wave 2, Years, Mean	11.65	11.72	*p* = .467
Age Wave 3, Years, Mean	12.98	13.16	*p* = .377

Table 1 summarizes the number of scans, medication usage, sex distribution, and mean age for ADHD and control groups across the 3 study time points, along with the results of between-group statistical tests.

ADHD, attention-deficit/hyperactivity disorder.

### Primary Analysis: Limbic System Volume Differences in Children With ADHD and Control Children

**Figure 2:**
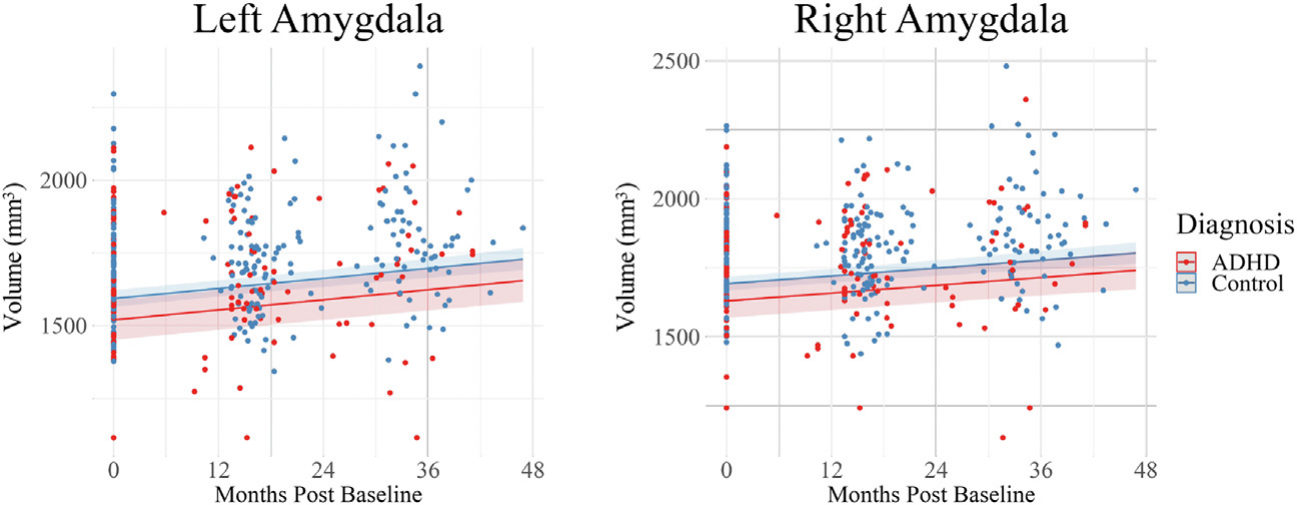
Amygdala volume growth across the 3 study time points. Bilateral amygdala development in the attention-deficit/hyperactivity disorder (ADHD) and control groups during the transition from childhood to adolescence. A group effect was observed in the amygdala (bilaterally); the ADHD group displayed lower volumes than the control group across the 3 time points in this study. No significant group-by-age interactions were observed in the amygdala (bilaterally).

**Figure 3:**
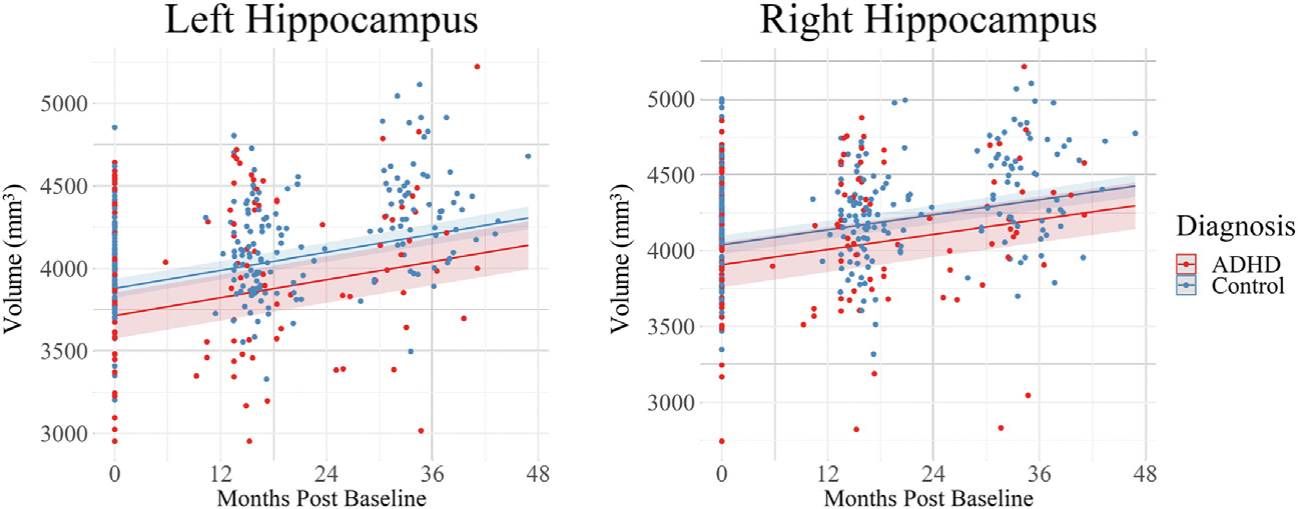
Hippocampus volume growth across the 3 study time points. Bilateral hippocampus development in the attention-deficit/hyperactivity disorder (ADHD) and control groups during the transition from childhood to adolescence. A group effect was observed in the hippocampus (bilaterally); the ADHD group displayed lower volumes than the control group across the 3 time points in this study. No significant group-by-age interactions were observed in the hippocampus (bilaterally).

**Figure 4:**
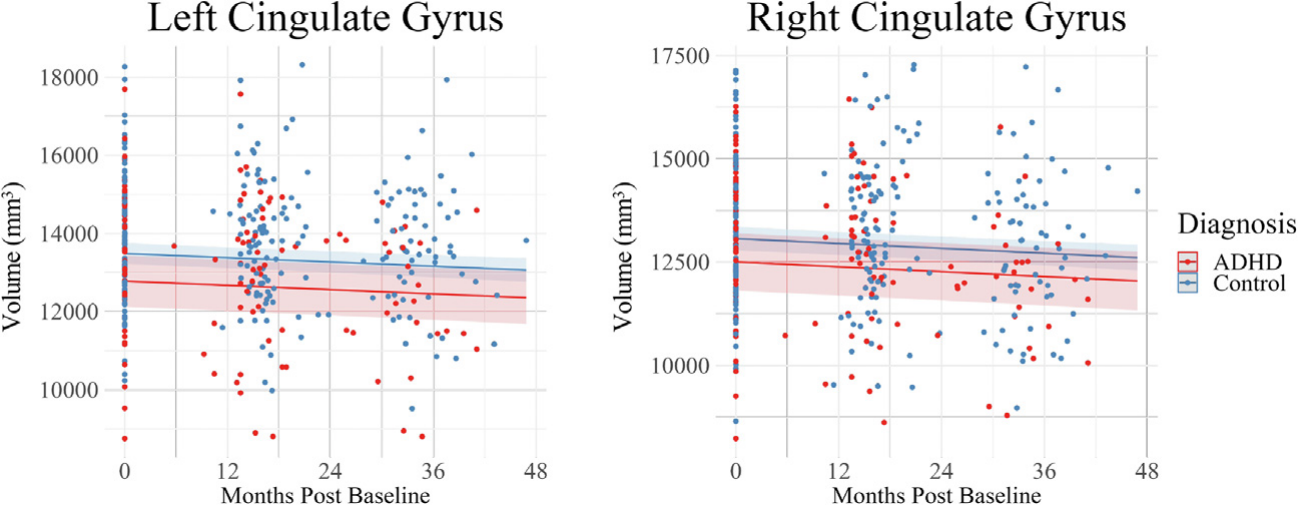
Cingulate gyrus volume growth across the 3 study time points. Bilateral cingulate gyrus development in the attention-deficit/hyperactivity disorder (ADHD) and control groups during the transition from childhood to adolescence. A group effect was observed in the cingulate gyrus (bilaterally); the ADHD group displayed lower volumes than the control group across the 3 time points in this study. No significant group-by-age interactions were observed in the cingulate gyrus (bilaterally).

**Figure 5:**
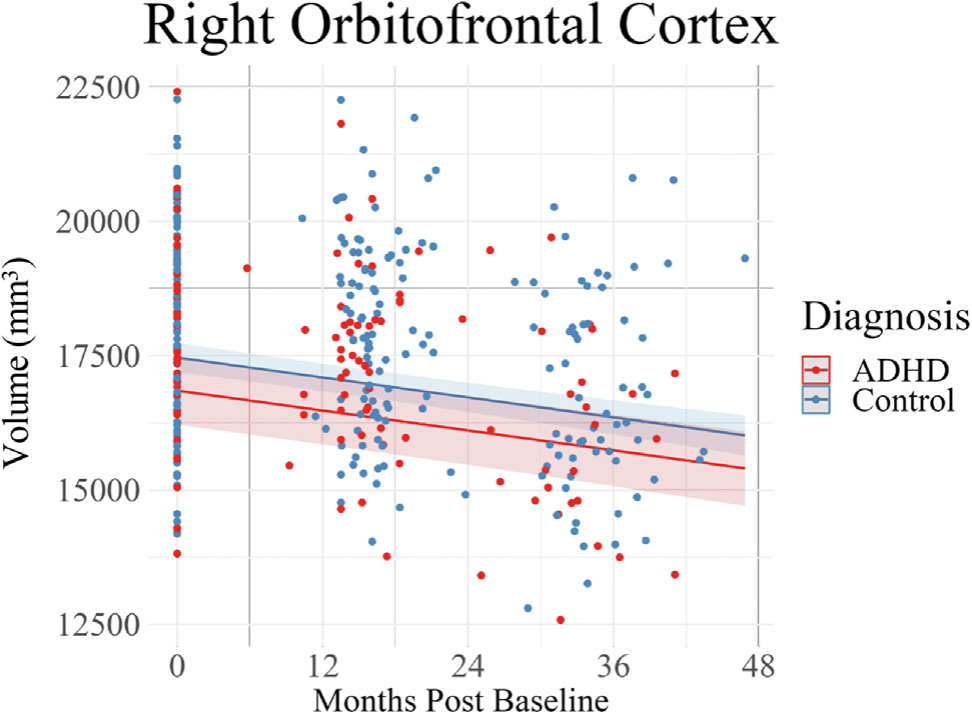
Right orbitofrontal cortex volume growth across the 3 study time points. Right orbitofrontal cortex development in the attention-deficit/hyperactivity disorder (ADHD) and control groups during the transition from childhood to adolescence. The ADHD group displayed lower volumes in the right orbitofrontal cortex than the control group across the 3 time points in this study. No significant group-by-age interactions were observed in the right orbitofrontal cortex.

There was a significant effect of group on volumes of the amygdala (left: β = −0.38, 95% CI, −0.66 to −0.11; right: β = −0.34, 95% CI, −0.60 to −0.08) (Figure 2), hippocampus (left: β = −0.44, 95% CI, −0.73 to −0.15; right: β = −0.34, 95% CI, −0.64 to −0.04) (Figure 3), cingulate gyrus (left: β = −0.42, 95% CI, −0.73 to −0.11; right: β = −0.32, 95% CI, −0.62 to −0.01) (Figure 4), and orbitofrontal cortex (right: β = −0.33, 95% CI, −0.59 to −0.06) (Figure 5). In all analyses across all 3 time points, there was lower volume of these structures in children with ADHD than in control children. All regions mentioned above survived 2-stage FDR correction. No significant difference in volume was observed between the groups for the bilateral mammillary bodies, anterior thalamic nuclei, or left orbitofrontal cortex (Figures S1–S3). There was no significant between-group difference in the group-by-age interaction bilaterally in the amygdala, hippocampus, cingulate gyrus, orbitofrontal gyrus, mammillary bodies, and anterior thalamic nuclei. See the Supplement for full results and model selection statistics (Tables S2–S5).

### Exploratory Analysis: The Relationship Between CAI and ARI Scores and Limbic System Volume in Children With ADHD

**Figure 6:**
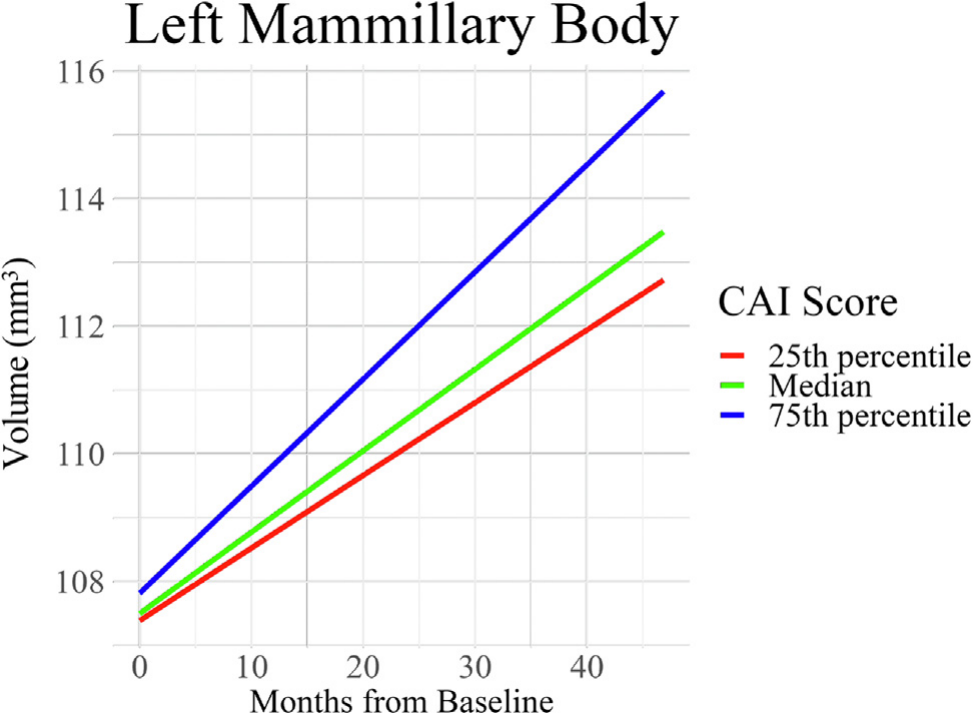
Effect of the interaction between attention-deficit/hyperactivity disorder (ADHD) symptom severity and age on left mammillary body volume in children and adolescents with ADHD. Associations between left mammillary body development and ADHD symptom severity (Conners 3 ADHD Index [CAI]) during the transition from childhood to adolescence within the ADHD group. As individuals with ADHD age, those with more severe symptoms tend to show a slower decline or even potential growth in left mammillary body volume than those with milder symptoms.

The exploratory analysis revealed a significant effect of the CAI-by-age interaction term on the volume of the left mammillary body (β_standardized = 0.17, 95% CI, 0.08 to 0.25) (Figure 6) in the ADHD group across the study time points. This finding survived 2-stage FDR correction. After adjusting for multiple comparisons, neither CAI nor ARI scores had any other significant effect on limbic system volumes in children and adolescents with ADHD (Tables S6, S7).

### Sensitivity Analyses

Sensitivity analyses suggested that the primary findings were robust to the possible confounding effects of sex balance across the groups (Table S8) and medication status (Tables S9–S13). Please refer to the Supplement for a comprehensive overview of the sensitivity analyses results.

## Discussion

In this longitudinal study, we investigated the volumetric development of limbic system structures in children and adolescents with ADHD. Across the 3 study time points, individuals with ADHD exhibited lower volume in the bilateral amygdala, hippocampus, cingulate gyrus, and right orbitofrontal cortex than control participants. Exploratory analysis identified a significant interaction between age and symptom severity on left mammillary body volume in the ADHD group, suggesting that limbic system development may play a role in the pathophysiology of ADHD. Consistent with the ENIGMA (Enhancing NeuroImaging Genetics through Meta Analysis) mega-analyses (15,45) and other recent studies (46,47), the sensitivity analysis revealed no significant association between structural brain changes and ADHD medication use in children and adolescents (Tables S12, S13).

### Between-Group Differences in the Development of Limbic System Structures

To further understand the findings of this study, it is helpful to consider the neurotypical developmental patterns of the brain structures that comprise the limbic system. The volume of cortical structures (e.g., orbitofrontal cortex and cingulate gyrus) increases rapidly from midgestation before peaking during childhood [orbitofrontal cortical volume peaks at age 6–7 years (48), and cingulate gyrus volume peaks at age 7–9 years (48)], followed by a near-linear decrease in volume from late childhood to late adulthood (48). This developmental pattern was observed in both control and ADHD groups in the cingulate gyrus (Figure 4) and orbitofrontal cortex (Figure 5) as evidenced by the volumetric decrease seen across the study time points. Despite the apparent typical developmental pattern, children with ADHD had persistently lower volumes of these structures than control children. These findings are consistent with the convergence model (14), which suggests that children with persistent ADHD symptoms display fixed, nonprogressive development of neural features throughout childhood and adolescence (14).

Subcortical structures (e.g., amygdala and hippocampus) increase in volume throughout childhood and early adolescence before attaining peak volume during midpuberty (14–15 years) (48,49), followed by a near-linear decrease in volume throughout adulthood (48). This developmental pattern was seen for both control and ADHD groups in the amygdala (Figure 2) and hippocampus (Figure 3) as indicated by the increase in the volumes of these structures across the study time points. Compared with the control group, however, the ADHD group showed persistently lower volume in these structures characterized by an apparent developmental lag. While it is possible that this finding reflects a developmental delay (11, 12, 13), longitudinal studies employing a wider age range that captures the age of peak volume attainment are crucial to investigate whether structures in an ADHD group would normalize later in development. Given that ADHD is associated with abnormalities across numerous brain structures with distinct developmental mechanisms and trajectories (48,49), the pathophysiology will likely involve varied region-specific developmental patterns. In response to reviewer comments, additional supplementary analyses were conducted investigating the between-group differences in regions that are key to ADHD, such as the basal ganglia, inferior prefrontal cortex, and dorsolateral prefrontal cortex (see Additional analyses: ADHD-associated brain regions in the Supplement).

Although the underlying mechanisms that lead to the lower volume and potentially delayed neurodevelopment in ADHD remain unknown, variants of several ADHD-associated genes have been shown to play a critical role in all stages of cortical development (50). A recent review (50) investigated how these ADHD-associated genes contribute to neurodevelopment and how variants in these genes could result in the neurologic phenotypes observed in the disorder. The most common effect of ADHD-associated genes on brain development is the disruption of synaptic formation and activity (50), and it has been suggested that lower gray matter volume in the brain may be due to the loss of synaptic density rather than neuronal cell loss (50). It has been proposed that variants in these ADHD-associated genes may contribute to the reduced gray matter seen in the disorder (50). Furthermore, the delayed establishment of neural connections—a process associated with ADHD susceptibility genes—has been shown to result in an underdeveloped brain (50), consistent with the patterns of reduced volumes in various brain structures seen in individuals with ADHD (50).

### Brain-Behavior Association Between Limbic System Volumes and ADHD Symptom Severity

While ADHD is characterized by age-related changes in symptoms and brain structure during childhood and adolescence, the link between brain structure and function is less clear. Exploratory analyses conducted in this study revealed a novel association between the developmental trajectory of left mammillary body volume and ADHD symptom severity. The analyses revealed a significant effect of age-by-symptom severity interaction on left mammillary body volume in the ADHD group. Based on the results of this study, as individuals with ADHD progress from childhood to adolescence, those with severe symptoms may show a slower decline or even a potential growth in left mammillary body volume than those with milder symptoms. This suggests that variations in mammillary body development may play a role in the persistence and increase of ADHD symptom severity during adolescence.

While there has been a long-standing awareness of the impact of mammillary body pathology in adults, researchers have only recently become aware of the significance of mammillary body pathology in younger populations (51). The mammillary bodies are an integral component of the limbic system, playing a pivotal role in encoding complex memories (52). While once thought to serve primarily as a relay to the hippocampus, recent studies have shown that through the limbic system pathways, the mammillary bodies influence a wide range of brain regions (52). The mammillary bodies have been shown to provide arousal and interoceptive information to boost and bias the iterative processing of the limbic system (52). As such, the input of the mammillary bodies can significantly affect emotional regulation, memory formation and recall, and behavior (52). It has been suggested that the mammillary bodies’ input plays a role in psychiatric and neurodevelopmental disorders like ADHD in which memory impairments and emotional dysregulation are commonly observed (52).

Mammillary body volumes are observed to increase until approximately 15 years old (53). Therefore, while individuals with increased symptom severity may seem to converge toward a normative pattern, the underlying functionality or efficiency of this region and its modulation of neural circuits, such as the limbic system, could still be compromised, thereby contributing to increased symptom severity. This finding underscores the belief that in ADHD, there may not only be differences in brain structure but also in how these structures modulate neural circuits to influence symptom expression (54). Structural MRI data provide limited insight into the functional dynamics of these changes. To fully understand the link between atypical mammillary body development and ADHD symptom severity, in-depth functional imaging and histological studies are essential.

The current study deployed a focused brain-behavior study approach (*n* = 57, total scans = 123) (55). This focused approach typically includes fewer participants but prioritizes improved classification of brain structure and behavior through the use of advanced MRI techniques, robust diagnostic classification, and measurement of within-subject variation in brain structure and behavior (55). Notably, this focused approach demonstrated the ability of smaller, focused sample approaches to yield similar effect sizes to those observed in large consortia studies (55). Both focused samples and large consortia studies play valuable roles in psychiatric research. Large consortia studies offer heightened statistical power and generalizability due to their larger sample sizes (56), while focused sample approaches, leveraging their precision and sensitivity, provide valuable insights into nuanced brain-behavior relationships (55).

### Brain-Behavior Association Between Limbic System Volumes and Emotional Dysregulation

While the findings of this study revealed a link between limbic system development and ADHD symptom severity, no significant association was found with emotional dysregulation. This may be partly because emotional regulation encompasses multilevel processes, including both bottom-up and top-down neural regulation (3). The limbic system incorporates bottom-up regulation, which involves the amygdala and orbitofrontal cortex. Bottom-up regulation is how individuals assess emotional stimuli and value rewards, allowing quick and automatic responses to emotional stimuli (3). It is worth considering that the presence of emotional dysregulation in ADHD is a complex cognitive process that is likely influenced by various neural circuits beyond the limbic system (3).

Top-down regulatory processing (which involves the ventrolateral prefrontal cortex, medial prefrontal cortex, and anterior cingulate gyrus) governs the allocation of attention and responses to emotional stimuli (57,58). This processing involves a more controlled and rational assessment of emotions, enabling individuals to modulate their reactions based on reasoned judgment (3). Because this study focused on the limbic system, the structures involved in top-down processing were not investigated. It is plausible that while the limbic system may play a role in emotional regulation, top-down processes may be more critical for emotional dysregulation in ADHD (3).

It is also possible that the metric used to measure emotion regulation in this study, the ARI, influenced the finding of a lack of association between emotion dysregulation and limbic system structures. Emotional dysregulation is a complex construct that can manifest in various forms, including problems with impulse control, mood swings, and an unusual fixation on emotional stimuli (3). Although the ARI is commonly used to measure emotional dysregulation, it may not fully capture this multifaceted construct (59). The ARI primarily measures irritability and reactivity, which are just 2 components of emotional dysregulation (59). It has been suggested that irritability refers to anger dysregulation whereas emotional dysregulation refers to dysregulation of both angry and positive emotions (59). As such, the ARI may provide only a partial representation of emotional dysregulation. Considering the complex and multidimensional nature of emotional regulation, future research on children with ADHD would benefit from incorporating a more comprehensive range of assessments, including but not limited to the Emotion Regulation Checklist (60), Temperament in Middle Childhood Questionnaire (61), and Children’s Emotion Management Scales (62, 63, 64).

### Limitations

A limitation of this study is that there were significantly fewer female than male participants at study time points 1 and 2. Despite well-characterized differences in symptom profiles between girls and boys with ADHD (65), sex-specific differences in brain development are less clear. Research has shown no difference between girls and boys with ADHD in developmental trajectories of brain volume (66) or cortical thickness (67). However, many studies that have investigated brain development in ADHD have not tested for sex differences, thus making it difficult to determine whether sex-specific differences in brain development are present in ADHD. The sensitivity analysis conducted in this study suggests that the sex imbalance between the ADHD and control groups in the sample did not significantly impact the results of the primary analysis (Table S8). Therefore, the findings of the primary analysis are unlikely to be confounded by this sex imbalance. Nevertheless, given that females with ADHD are often underrepresented in case-control studies, there is a need for future research to include a sufficient number of female participants to investigate sex-specific differences in the disorder.

Another limitation of this study is that the presence or absence of comorbidities in ADHD was not recorded during data collection. Data on behavioral disorders would have been of particular interest given that the focus of this study was on emotional dysregulation. This study also did not specifically investigate ADHD subtypes (inattentive, hyperactive/impulsive, and combined). While many of the smaller sample-sized studies (*n* < 50) failed to identify differences in volume between subtypes (68, 69, 70), larger-scale studies have now suggested that there may be subtle volumetric differences across the subtypes (71,72). Therefore, it is important that future research investigate developmental differences across ADHD subtypes.

### Conclusions

In this longitudinal study, we found that compared with control participants, children and adolescents with ADHD had lower gray matter volume across development in key limbic system structures. Additionally, among individuals with ADHD, the developmental trajectory of the left mammillary body was significantly associated with alterations in symptom severity during the transition from childhood into adolescence. Taken together, these findings indicate that atypical development in limbic system structures appears to be a potential neurobiological feature of ADHD, which advances our understanding of the pathophysiology of this highly prevalent neurodevelopmental disorder.

## Supplementary Material

Supplement

Key Resources Table
