# Supplementary material for: The Limbic System in Children and Adolescents With Attention-Deficit/Hyperactivity Disorder: A Longitudinal Structural Magnetic Resonance Imaging Analysis
Source: Biol Psychiatry Glob Open Sci. 2023 Nov 2;4(1):385–93. doi: 10.1016/j.bpsgos.2023.10.005 (PMC10829648; doi:10.1016/j.bpsgos.2023.10.005)
Supplement: Supplement [file mmc1.pdf]

SUPPLEMENTARY INFORMATION

The Limbic System in Children and Adolescents With Attention-Deficit/Hyperactivity Disorder: A Longitudinal Structural Magnetic Resonance Imaging Analysis

Connaughton *et al.*

Supplemental Material

Table of Contents

1. **Manual image quality control procedure** 1

2. **Primary Analysis: Limbic system volume differences in children with ADHD and controls** 1

Top-down Model Selection Procedure 1

Model Selection Results: Limbic Lobe Structures in ADHD and Controls 5

Sensitivity Analysis 1: 15

Sensitivity Analysis 2: 18

3. **Additional analyses: ADHD-associated brain regions** 23

Inferior Prefrontal Cortex 23

Dorsolateral Prefrontal Cortex 29

Thalamus 33

Basal Ganglia 35

1. Manual image quality control procedure

Raw T1 and T2 images were rated on a 4-point Likert scale: 1) sharply defined image and/or no ringing artifacts; 2) slight blurriness and/or slight ringing limited to a small cortical area; 3) considerable blurriness and/or considerable ringing throughout more of the brain extending into white matter regions; 4) extensive blurriness and/or ringing across the entire brain. This manual quality control check was conducted on the full data set. Any image that received a score of >3 was excluded from this study (N=7).

2. Primary Analysis: Limbic system volume differences in children with ADHD and controls

Top-down Model Selection Procedure

The details of the LME models tested in this study are presented in Table 1. IQ was not included as a covariate in any of the tested models as the inclusion of IQ is deemed inappropriate for neurodevelopmental disorders involving cognitive deficits such as ADHD as it can lead to overcorrected and spurious findings(1). Starting with the most complex model, random and fixed effects were removed in a backward fashion using a combination of fit statistics – corrected Akaike Information Criterion (AICc), corrected Bayesian Information Criterion (BICc), and log-likelihood ratio test (LRT) – to identify the optimal model for each limbic lobe structure. Random effects were identified by comparing the fit of the models with and without the random effect of slope (RX1a vs. RX1b). The random effect of slope was included if it significantly increased the model fit and the model's boundary was not singular. The fixed effects of interest (i.e., group and group-by-age interaction) were identified by comparing the model fit of the null model (a model that only contained the covariate fixed effects: age at baseline, age, sex, and intracranial volume) against both fixed-effects models (FX1/FX2 vs. null0). If a fixed-effects model significantly increased fit compared to the null model, this indicated a significant effect on volume due to the fixed effects of interest. If both fixed effects models significantly increased model fit compared to the null model, but there was no difference in model fit between the two fixed effects models (FX1 vs. FX2), the parsimonious model (FX1) was selected (i.e., including the interaction term was not justified). The results of the model fit statistics for each region are presented in eTables 1 and 2. When initially comparing model fit, maximum likelihood (ML) was used to allow for comparability with the model fit parameters AICc and BICc. However, once the "optimal model" was identified, this model was refitted using Restricted Maximum Likelihood (REML) to increase the accuracy of estimates of the final model parameters. The covariance structure was set to "unstructured", which is the default covariance structure in R software.

This structure imposes no restrictions on the covariance parameters, thus allowing the inclusion of variations in the slopes.

eTable 1. Linear mixed Models Tested: Limbic Lobe Structures in ADHD and Controls

Random Effects Models	
RX 1a	ROI ~ ICV + Age at baseline + sex + diagnosis*age + (1 + age |subject)
RX 1b	ROI ~ ICV + Age at baseline + sex + diagnosis* age + (1|subject)
Fixed Effects Models	
Null 0a	ROI ~ ICV + age + Age at baseline + sex + (1 + age | subject)
Null 0b	ROI ~ ICV + age + Age at baseline + sex + (1|subject)
FX 1a	ROI ~ ICV + sex + age + Age at baseline + diagnosis + (1 + age |subject)
FX 1b	ROI ~ ICV + sex + age + Age at baseline + diagnosis + (1|subject)
FX 2a	ROI ~ ICV + sex + age + Age at baseline + diagnosis* age + (1 + age |subject)
FX 2b	ROI ~ ICV + sex + age + Age at baseline + diagnosis* age + (1|subject)
FX 3a	ROI ~ ICV + sex + age*ADHD symptom + (1|subject)

Note: RX = random effects, FX = fixed effects, ROI = regions of interest, ICV = intracranial volume, age = participant age from baseline (in months). To increase iterability, the variables ICV and age were mean-centered at baseline. ADHD symptom = CAI and ARI score

eTable 2. Fit indices of random effects: Limbic Lobe Structures in ADHD and Controls

**Region of Interest**	**Model**	**AICc**	**BICc**	**Log-likelihood**	**Test**	**LRT (p-value)**
Hippocampus (left)*****	1a	5145.48	5149.43	-2578.82		
	1b	5147.32	5151.27	-2584.38	1a vs 1b	0.398
Hippocampus (right)*****	1a	5173.37	5177.32	-2597.72		
	1b	5174.81	5178.76	-2598.44	1a vs 1b	0.486
Amygdala (left)	1a	4727.76	4731.71	-2370.49		

	1b	4729.01	4732.96	-2370.98	1a vs 1b	0.534
Amygdala (right)	1a	4650.88	4654.84	-2331.48		
	1b	4665.3	4669.25	-2338.49	1a vs 1b	**< 0.001**
Cingulate gyrus (left)	1a	6119.58	6123.53	-3080.46		
	1b	6132.42	6136.37	-3086.61	1a vs 1b	**0.001**
Cingulate gyrus (right)	1a	6173.67	6177.63	-3107.92		
	1b	6182.24	6186.19	-3111.98	1a vs 1b	**0.013**
Orbitofrontal cortex (left)*****	1a	6413.55	6417.50	-3229.06		
	1b	6414.27	6418.23	-3229.41	1a vs 1b	0.696
Orbitofrontal cortex (right)	1a	6446.70	6450.66	-3245.87		
	1b	6447.12	6451.07	-3246.03	1a vs 1b	0.813
Anterior thalamic nuclei (left)	1a	3030.19	3034.13	-1505.87		
	1b	3033.01	3036.95	-1507.21	1a vs 1b	0.244
Anterior thalamic nuclei (right)	1a	3005.53	3009.47	-1493.26		
	1b	3008.75	3012.69	-1494.78	1a vs 1b	0.200
Mammillary bodies (left)	1a	2789.37	2793.32	-1383.16		
	1b	2793.76	2797.71	-1385.19	1a vs 1b	0.111
Mammillary bodies (right)	1a	2801.06	2805.01	-1389.17		
	1b	2805.27	2809.21	-1391.26	1a vs 1b	0.121

Note: LRT = likelihood-ratio test. Bold indicates selected random effects model. AIC = Akaike Information Criterion. BIC = Bayesian Information Criterion. *model is singular

eTable 3. Fit indices of fixed effects: Limbic Lobe Structures in ADHD and Controls

**Region of Interest**	**Model**	**AICc**	**BICc**	**Log-likelihood**	**Test**	**LRT (p-value)**
Hippocampus (left)	0b	5164.35	5168.30			
	1b	5145.99	5149.94	-2582.57	0 vs 1	**0.010**
	2b	5137.98	5141.95	-2579.62	0 vs 2	0.029
					1 vs 2	0.499
Hippocampus (right)	0b	5161.77	5165.73			
	1b	5147.7	5151.65	-2583.59	0 vs 1	**0.027**
	2b	5145.34	5149.30	-2583.48	0 vs 2	0.073
					1 vs 2	0.566
Amygdala (left)	0b	4725.42	4729.38			
	1b	4710.27	4714.22	-2360.81	0 vs 1	**0.006**
	2b	4707.51	4711.46	-2360.03	0 vs 2	0.009
					1 vs 2	0.162
Amygdala (right)	0a	4635.80	4639.76			
	1a	4621.3	4625.25	-2315.82	0 vs 1	**0.010**
	2a	4619.86	4623.82	-2315.68	0 vs 2	0.029
					1 vs 2	0.499
Cingulate gyrus (left)	0a	6136.04	6139.99			
	1a	6116.07	6120.02	-3076.24	0 vs 1	**0.027**
	2a	6111.15	6115.10	-3076.16	0 vs 2	0.073
					1 vs 2	0.566
Cingulate gyrus (right)	0a	6199.62	6203.58			
	1a	6182.25	6186.21	-3109.81	0 vs 1	**0.006**
	2a	6176.50	6180.46	-3109.28	0 vs 2	0.009

					1 vs 2	0.162
Orbitofrontal cortex (left)	0b	6440.92	6444.87			
	1b	6423.51	6427.46	-3231.12	0 vs 1	0.030
	2b	6413.71	6417.67	-3229.13	0 vs 2	**0.013**
					1 vs 2	0.048
Orbitofrontal cortex (right)	0b	6477.4	6481.35			
	1b	6458.51	6462.47	-3248.76	0 vs 1	**0.013**
	2b	6450.30	6454.26	-3247.66	0 vs 2	0.014
					1 vs 2	0.124
Anterior thalamic nuclei (left)	0a	2993.57	2997.52			
	1a	2995.15	2999.09	-1489.6	0 vs 1	0.575
	2a	2997.06	3001.01	-1488.92	0 vs 2	0.443
					1 vs 2	0.251
Anterior thalamic nuclei (right)	0b	3000.52	3004.47			
	1b	2995.93	2999.87	-1489.81	0 vs 1	0.447
	2b	2998.23	3002.17	-1489.38	0 vs 2	0.477
					1 vs 2	0.341
Mammillary bodies (left)	0b	2791.43	2795.38			
	1b	2787.59	2791.53	-1383.96	0 vs 1	0.395
	2b	2791.32	2795.27	-1383.95	0 vs 2	0.696
					1 vs 2	0.979
Mammillary bodies (right)	0b	2817.43	2821.38			
	1b	2813.92	2817.87	-1397.61	0 vs 1	0.655
	2b	2816.61	2820.56	-1398.03	0 vs 2	0.517
					1 vs 2	0.290

Model Selection Results: Limbic Lobe Structures in ADHD and Controls

Random effects structure:

Models that included intercept and slope as random effects were found to be the best fit for the amygdala (right) and cingulate gyrus (bilateral). The model that included only the intercept as a random effect was the best fit for the amygdala (left), hippocampus (bilateral), mammillary body (bilateral), anterior thalamic nuclei (bilateral), and orbitofrontal cortex (bilateral).

Fixed effects structures:

The model that included both fixed effects (i.e., group-by-age interaction) was the best fit for the orbitofrontal cortex (left). The reduced model that only included the fixed effect of group was the best fit for the amygdala (bilateral), hippocampus (bilateral),

orbitofrontal cortex (right), and cingulate gyrus (bilateral). For the mammillary bodies (bilateral) and anterior thalamic nuclei (bilateral), no significant difference in model fit was found between

the fixed effects models and the null model, implying that in these structures, the null hypothesis could not be rejected due to the fixed effects of interest.

eTable 4. Results of optimal mixed-effects models (without interaction term) analyses: Limbic Lobe Structures in ADHD and Controls.

	**ICV**		**Sex**		**Months from baseline**		**Age at baseline**		**Diagnosis**	
Region of Interest	***B*** **(SE)**	**t, p**	***B*** **(SE)**	**t, p**	***B*** **(SE)**	**t, p**	***B*** **(SE)**	**t, p**	***B*** **(SE)**	**t, p**
Hippocampus (Left)	4.384e-05 (7.986e-05)	0.549, 0.583	2.097e+02 (5.356e+01)	3.915, <0.000	9.093e+00 (5.875e-01)	15.476, <0.000	4.771e+01 (5.535e+01)	0.862, 0.389	-1.655e+02 (5.533e+01)	-2.991, **0.003** (0.028)*
Hippocampus (Right)	7.171e-05 (8.111e-05)	0.884, 0.377	1.501e+02 (5.678e+01)	2.643, 0.008	8.295e+00 (5.926e-01)	13.998, <0.000	3.665e+01 (5.894e+01)	0.622, 0.535	-1.298e+02 (5.902e+01)	-2.200, **0.029** (0.040)*
Amygdala (Left)	3.786e-05 (4.793e-05)	0.790, 0.430	1.557e+02 (2.688e+01)	5.792, <0.000	2.887e+00 (3.670e-01)	7.865, <0.000	3.474e+01 (2.734e+01)	1.271, 0.205	-7.368e+01 (2.716e+01)	-2.713, **0.007** (0.028)*
Amygdala (Right)	6.040e-05 (4.406e-05)	1.371, 0.171	1.477e+02 (2.393e+01)	6.174, <0.000	2.362e+00 (3.769e-01)	6.268, <0.000	5.469e+00 (2.451e+01)	0.223, 0.823	-6.244e+01 (2.429e+01)	-2.570 **0.011** (0.034)*
Cingulate Gyrus (Left)	5.827e-04 (2.656e-04)	2.194, 0.029	6.041e+02 (2.358e+02)	2.562, 0.011	-9.063e+00 (2.098e+00)	-4.320, <0.000	-9.060e+01 (2.575e+02)	-0.352, 0.725	-7.073e+02 (2.619e+02)	-2.700, **0.007** (0.028)*
Cingulate Gyrus (Right)	1.511e-03 (2.880e-04)	5.248, <0.000	4.021e+02 (2.510e+02)	1.602, 0.110	-9.859e+00 (2.231e+00)	-4.419, <0.000	-2.778e+02 (2.720e+02)	-1.022, 0.308	-5.651e+02 (2.764e+02)	-2.044, **0.042** (0.043)*
Orbitofrontal Cortex (Right)	1.973e-03 (4.974e-04)	3.967, <0.000	1.008e+03 (2.513e+02)	4.011, <0.000	-3.095e+01 (3.944e+00)	-7.846, <0.000	3.735e+02 (2.535e+02)	1.473, 0.142	-6.151e+02 (2.510e+02)	-2.451, **0.015** (0.037)*

* = Factors that survived Two-stage FDR correction, corrected p is reported below.

eTable 5. Results of optimal mixed-effects models (with interaction term) analyses: Limbic Lobe Structures in ADHD and Controls.

	**ICV**		**Sex**		**Months from baseline**		**Age at baseline**		**Diagnosis**		**Diagnosis * Months from baseline**	
Region of Interest	***B*** **(SE)**	**t, p**	***B*** **(SE)**	**t, p**	***B*** **(SE)**	**t, p**	***B*** **(SE)**	**t, p**	***B*** **(SE)**	**t, p**	***B*** **(SE)**	**t, p**
Orbitofrontal Cortex (Left)	1.905-03 (4.712e-04)	4.043, <0.000	8.154e+02 (2.503e+02)	3.258, 0.001	-2.495e+01 (3.997e+00)	-6.240, <0.000	1.024e+02 (2.533e+02)	0.404, 0.686	-3.597e+02 (2.679e+02)	-1.343, 0.180	-1.495e+01 (7.647e+00)	-1.955, 0.051
Anterior Thalamic Nuclei (Left)	1.453e-05 (5.163e-06)	2.815, 0.005	3.193e+00 (2.757e+00)	1.158, 0.248	4.410e-02 (4.383e-02)	1.006, 0.315	-5.949e-01 (2.803e+00)	-0.212, 0.832	-3.702e-01 (2.951e+00)	-0.125, 0.900	-9.513e-02 (8.386e-02)	-1.134, 0.257
Anterior Thalamic Nuclei (Right)	1.730e-05 (5.032e-06)	3.438, <0.000	3.822e+00 (2.596e+00)	-0.923, 0.357	-3.994e-02 (4.327e-02)	-0.923, 0.357	-7.995e-01 (2.632e+00)	-0.304, 0.761	-9.979e-01 (2.786e+00)	-0.358, 0.720	-7.791e-02 (8.289e-02)	-0.940, 0.348
Mammillary Bodies (Left)	3.347e-06 (3.915e-06)	0.855, 0.393	5.88e+00 (1.912e+00)	2.819, 0.005	1.392e-01 (3.214e-02)	4.330, <0.000	-9.846e-01 (1.912e+00)	-0.515, 0.607	-1.578e+00 (2.037e+00)	-0.775, 0.439	-9.730e-04 (6.157e-02)	-0.016, 0.987
Mammillary Bodies (Right)	2.338e-06 (3.899e-06)	0.600, 0.549	2.189e+00 (2.101e+00)	1.042, 0.299	2.126e-01 (3.076e-02)	6.911 <0.000	2.506e+00 (2.120e+00)	1.182, 0.239	-1.859e-01 (2.225e+00)	-0.084, 0.933	-6.136e-02 (5.872e-02)	-1.045, 0.297

eFigure 1. Left orbitofrontal cortex volume growth across the three study time points.

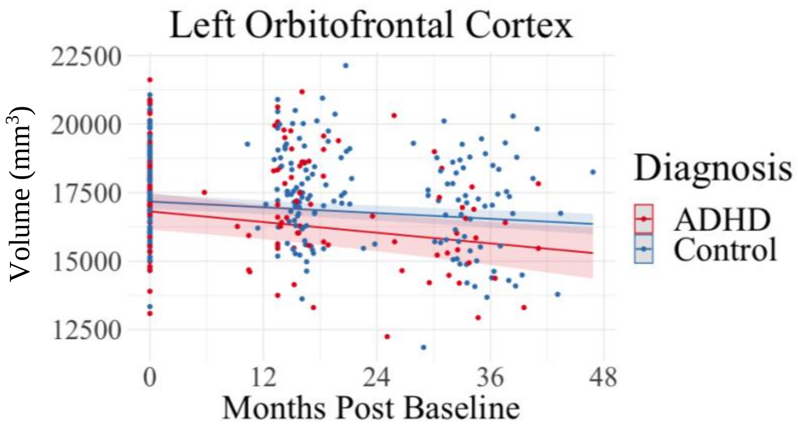

eFigure 2. Bilateral mammillary body volume growth across the three study time points.

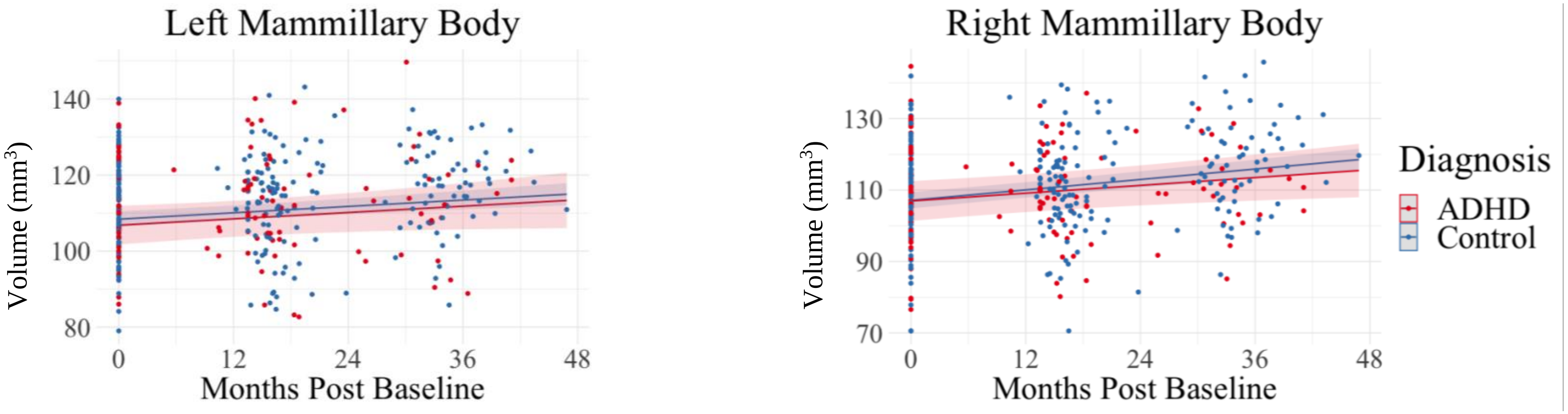

eFigure 3. Bilateral anterior thalamic nuclei volume growth across the three study time points.

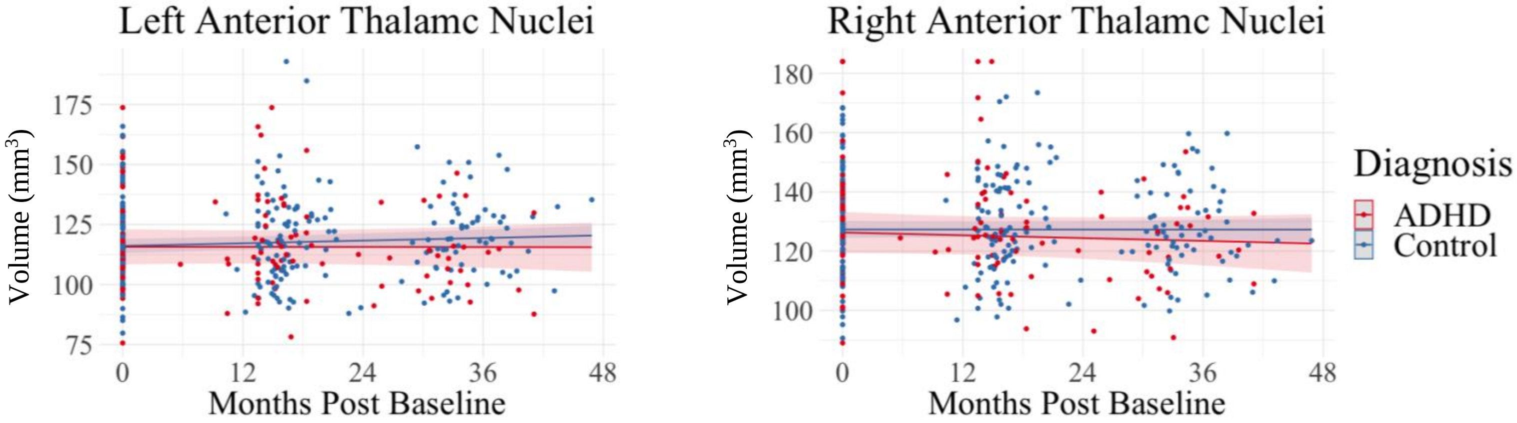

eTable 6. Results of mixed-effects models (with interaction term) analyses: limbic lobe network volume and CAI scores in ADHD.

	**ICV**		**Sex**		**Age**		**CAI**		**Age * CAI**	
Region of Interest	***B* (SE)**	**t, p**	***B* (SE)**	**t, p**	***B* (SE)**	**t, p**	***B* (SE)**	**t, p**	***B* (SE)**	**t, p**
Hippocampus (Left)	-1.064e-05 (1.267e-04)	-0.084, 0.933	5.940e+02 (1.234e+02)	4.813, <0.000	7.326e+00 (2.448e+00)	2.993, 0.004	-4.095e+00 (4.632e+00)	-0.884, 0.379	-2.776e-02 (1.730e-01)	-0.160, 0.873
Hippocampus (Right)	1.143e-04 (1.438e-04)	0.796, 0.429	4.505e+02 (1.256e+02)	3.587, <0.000	7.917e+00 (2.838e+00)	2.789, 0.007	-3.554e+00 (5.265e+00)	-0.675, 0.501	-4.201e-02 (2.008e-01)	-0.209, 0.835
Amygdala (Left)	4.311e-05 (7.308e-05)	0.590, 0.556	2.738e+02 (5.406e+01)	5.064, <0.000	-6.093e-01 (1.513e+00)	-0.403, 0.688	-3.146e+00 (2.687e+00)	-1.171, 0.244	2.154e-01 (1.073e-01)	2.006, 0.049
Amygdala (Right)	9.652e-05 (6.281e-05)	1.537, 0.128	2.469e+02 (4.739e+01)	5.210, <0.000	2.019e+00 (1.291e+00)	1.564, 0.123	1.804e-02 (2.308e+00)	0.008 0.994	2.023e-02 (9.159e-02)	0.221, 0.826
Cingulate Gyrus (Left)	3.571e-04 (4.660e-04)	0.766, 0.446	1.404e+03 (5.604e+02)	2.505, 0.015	-1.073e+01 (8.750e+00)	-1.226, 0.225	-3.138e+01 (1.700e+01)	-1.846, 0.069	-1.781e-01 (6.172e-01)	-0.289, 0.773
Cingulate Gyrus (Right)	1.874e-03 (4.694e-04)	3.991, <0.000	5.378e+02 (5.649e+02)	0.952, 0.345	-5.024e+00 (8.814e+00)	-0.570, 0.571	-2.213e+01 (1.712e+01)	-1.293, 0.200	-7.817e-01 (6.217e-01)	-1.257, 0.213
Orbitofrontal Cortex (Left)	1.677e-03 (7.349e-04)	2.282, 0.024	1.041e+03 (5.445e+02)	1.912, 0.061	-5.097e+01 (1.521e+01)	-3.352, 0.001	6.317e+01 (2.702e+01)	-2.337, 0.021	1.219e+00 (1.079e+00)	1.130, 0.263
Orbitofrontal Cortex (Right)	1.855e-03 (7.714e-04)	2.405, 0.017	1.024e+03 (5.023e+02)	2.038, 0.046	-2.894e+01 (1.690e+01)	-1.713, 0.091	-1.599e+01 (2.855e+01)	-0.560, 0.576	-1.193e+00 (1.202e+00)	-0.993, 0.324
Anterior Thalamic Nuclei (Left)	6.548e-06 (8.469e-06)	0.773, 0.441	7.905e-01 (6.131e+00)	0.129, 0.898	2.037e-01 (1.768e-01)	1.152, 0.254	4.005e-01 (3.117e-01)	1.285, 0.202	-1.614e-02 (1.255e-02)	-1.287, 0.203
Anterior Thalamic Nuclei (Right)	6.548e-06 (8.469e-06)	0.773, 0.441	7.905e-01 (6.131e+00)	0.129, 0.898	2.037e-01 (1.768e-01)	1.152, 0.254	4.005e-01 (3.117e-01)	1.285, 0.202	-1.614e-02 (1.255e-02)	-1.287, 0.203
Mammillary Body (Left)	-4.143e-06 (5.186e-06)	-0.799, 0.426	9.872e+00 (3.985e+00)	2.477, 0.016	-2.404e-01 (1.060e-01)	-2.268, 0.026	-2.230e-01 (1.905e-01)	-1.171, 0.244	2.775e-02 (7.514e-03)	3.693, **<0.000**

Mammillary Body (Right)	5.140e-07 (5.971e-06)	0.086, 0.931	1.061e+01 (4.279e+00)	2.480, 0.016	5.295e-03 (1.251e-01)	0.042, 0.966	-7.715e-02 (2.198e-01)	-0.351, 0.726	1.449e-02 (8.883e-03)	1.631, 0.108

eTable 7. Results of mixed-effects models (with interaction term) analyses: limbic lobe network volume and ARI scores in ADHD.

	**ICV**		**Sex**		**Age**		**ARI**		**Age * ARI**	
Region of Interest	***B* (SE)**	**t, p**	***B* (SE)**	**t, p**	***B* (SE)**	**t, p**	***B* (SE)**	**t, p**	***B* (SE)**	**t, p**
Hippocampus (Left)	4.578e-04 (2.033e-04)	2.252, 0.272	5.388e+02 (1.103e+02)	4.886, <0.000	1.477e+00 (2.326e+00)	0.635, 0.529	4.337e+00 (8.679e+00)	0.500, 0.619	3.218e-01 (4.001e-01)	0.804, 0.426
Hippocampus (Right)	8.497e-04 (2.226e-04)	3.817, 0.001	3.549e+02 (1.091e+02)	3.253, 0.002	2.405e+00 (2.779e+00)	0.866, 0.392	3.622e+00 (9.782e+00)	0.370, 0.712	2.617e-01 (4.735e-01)	0.553, 0.583
Amygdala (Left)	2.555e-04 (1.150e-04)	2.221, 0.029	2.349e+02 (5.325e+01)	4.411, <0.000	-8.680e-01 (1.537e+00)	-0.565, 0.576	2.905e+00 (5.164e+00)	0.563, 0.575	2.676e-01 (2.601e-01)	1.029, 0.309
Amygdala (Right)	3.195e-04 (9.138e-05)	3.497, <0.000	2.217e+02 (4.361e+01)	5.084, <0.000	1.190e+00 (1.174e+00)	1.013, 0.317	3.173e+00 (4.053e+00)	0.783, 0.436	4.800e-03 (1.995e-01)	0.024, 0.980
Cingulate Gyrus (Left)	3.943e-03 (7.190e-04)	5.484, <0.000	9.801e+02 (4.864e+02)	2.015, 0.049	1.138e+01 (7.375e+00)	-1.544, 0.131	-6.138e+00 (2.951e+01)	-0.208, 0.836	9.506e-01 (1.286e+00)	0.739, 0.464
Cingulate Gyrus (Right)	4.480e-03 (8.050e-04)	5.566, <0.000	2.021e+02 (5.334e+02)	0.379, 0.706	1.848e+00 (8.318e+00)	0.222, 0.825	-1.961e-01 (3.313e+01)	-0.006, 0.995	-2.094e+00 (1.449e+00)	-1.445, 0.156
Orbitofrontal Cortex (Left)	4.954e-03 (1.057e-03)	4.688, <0.000	3.766e+02 (4.799e+02)	0.785, 0.436	-2.616e+00 (1.452e+01)	-0.180, 0.858	-7.794e+01 (4.784e+01)	-1.629, 0.107	-1.463e+00 (2.450e+00)	-0.597, 0.553
Orbitofrontal Cortex (Right)	6.153e-03 (9.836e-04)	6.255, <0.000	2.379e+02 (4.073e+02)	0.584, 0.562	-3.542e+01 (1.629e+01)	-2.174, 0.035	-1.134e+02 (4.702e+01)	-2.412, 0.018	1.551e+00 (2.699e+00)	0.574, 0.568
Anterior Thalamic Nuclei (Left)	2.552e-05 (1.393e-05)	1.833, 0.070	-8.832e-01 (6.038e+00)	-0.146, 0.884	-1.974e-02 (2.074e-01)	-0.095, 0.924	-4.760e-02 (6.458e-01)	-0.074, 0.941	-5.770e-04 (3.473e-02)	-0.017, 0.986
Anterior Thalamic Nuclei (Right)	2.552e-05 (1.393e-05)	1.833, 0.070	-8.832e-01 (6.038e+00)	-0.146, 0.884	-1.974e-02 (2.074e-01)	-0.095, 0.924	-4.760e-02 (6.458e-01)	-0.074, 0.941	-5.770e-04 (3.473e-02)	-0.017, 0.986
Mammillary Body (Left)	1.321e-05 (8.443e-06)	1.565, 0.121	8.006e+00 (4.050e+00)	1.977, 0.053	-1.917e-01 (1.079e-01)	-1.777, 0.082	-2.225e-01 (3.737e-01)	-0.595, 0.553	4.039e-02 (1.834e-02)	2.203, 0.032

Mammillary Body (Right)	2.493e-05 (8.903e-06)	2.800, 0.006	7.935e+00 (4.098e+00)	1.936, 0.058	-2.251e-01 (1.200e-01)	-1.876, 0.067	-9.168e-02 (4.007e-01)	-0.229, 0.819	5.415e-02 (2.028e-02)	2.670, 0.010

Sensitivity Analysis 1:

The first sensitivity analysis examined the potential confound caused by the case-control sex imbalance in the study sample. To investigate this, 100 LME iterations were run using randomly selected proportionately sex-matched case-control samples. To ensure the sex-matching in the control group was unbiased, 65 female controls scans were randomly excluded at each iteration using a script developed in R studio (v.4.1.1). Averages of all 100 iterations were then collected to compare the results of the sensitivity analysis to the primary analysis. In the 100 random sex-matched analyses, there was a slight reduction in p-value significance. This was expected given the increases in standard errors (SE) related to the reduction in sample size (eTable 8). The right hippocampus was the only structure that no longer survived post hoc analysis following sensitivity analysis. To further understand the impact of group sex balance on right hippocampus volume, we compared beta weights and SEs of the main analysis and sex-balanced sensitivity models. Importantly, in the right hippocampus models, the beta values for the main effect of diagnosis were still within the SEs of the optimal models (see eFigure 4), demonstrating that the overall pattern of results in the primary analysis was most likely not confounded by a sex-ratio group imbalance in the study.

eTable 8. Mean values of the effect of Diagnosis in the 100 iterations of optimal mixed-effects models analyses with sex sex-matched case-control samples

	Diagnosis			
Region of Interest	***B (SD)***	**SE (SD)**	***T (SD)***	***p* (SD)**
Hippocampus (Left)	-151.125<br>(9.548)	58.257<br>(0.857)	-2.594<br>(0.164)	**0.012***<br>(0.005)
Hippocampus (Right)	-120.955<br>(9.921)	61.591<br>(0.958)	-1.964<br>(0.162)	0.055<br>(0.020)
Amygdala (Left)	-70.055<br>(4.370)	28.397<br>(0.500)	-2.467<br>(0.142)	**0.016***<br>(0.006)
Amygdala (Right)	-61.282<br>(2.764)	24.679<br>(0.246)	-2.483<br>(0.108)	**0.015***<br>(0.005)
Cingulate Gyrus (Left)	-710.609<br>(51.894)	268.437<br>(5.313)	-2.648<br>(0.195)	**0.010***<br>(0.005)
Cingulate Gyrus (Right)	-583.268<br>(42.539)	283.561<br>(4.067)	-2.057<br>(0.152)	**0.044***<br>(0.015)
Orbitofrontal Cortex (Left)	-343.072<br>(49.202)	269.478<br>(6.215)	-1.275<br>(0.193)	0.212<br>(0.070)
Orbitofrontal Cortex (Right)	-622.017<br>(40.934)	254.495<br>(5.094)	-2.445<br>(0.174)	**0.017***<br>(0.008)
Anterior thalamic nuclei (Left)	-0.574<br>(0.490)	3.041<br>(0.061)	-0.188<br>(0.161)	0.839<br>(0.106)
Anterior thalamic nuclei (Right)	-1.418<br>(0.435)	2.921<br>(0.042)	-0.486<br>(0.151)	0.632<br>(0.105)
Mammillary bodies (Left)	-1.243<br>(0.357)	2.108<br>(0.027)	-0.590<br>(0.169)	0.562<br>(0.114)
Mammillary bodies (Right)	0.154<br>(0.468)	2.306<br>(0.037)	0.068<br>(0.203)	0.860<br>(0.092)

SD = standard deviations, * = survived Two-stage FDR correction.

eFigure 4: Beta value and standard errors of the main effect diagnosis in the sex-balanced sensitivity analysis

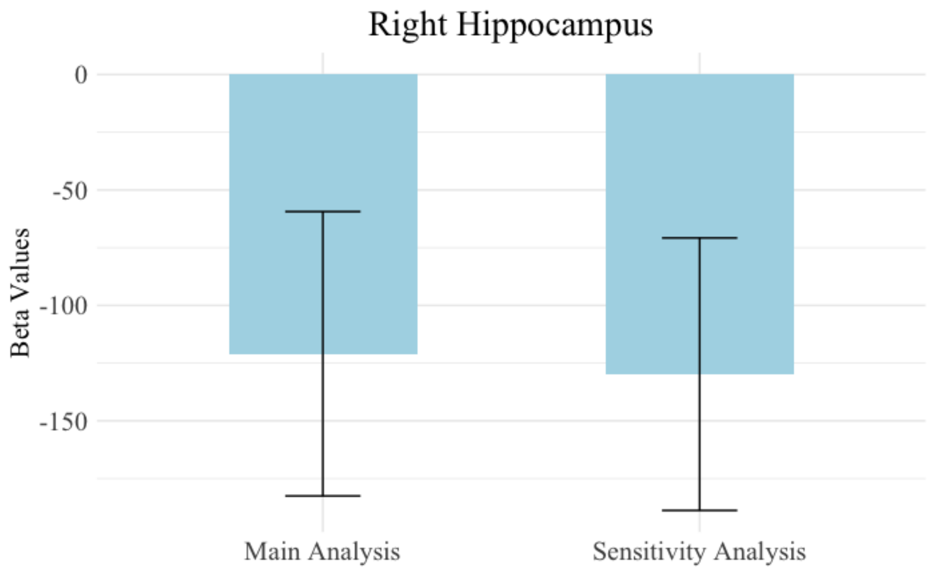

Sensitivity Analysis 2:

The second sensitivity analysis explored the potential impact of medication use in the ADHD group by conducting LME analyses comparing individuals with ADHD who were taking medication and those who were not. The methodology and results of the model selection procedure are provided (eTable 9-11). The results of the second sensitivity analysis revealed no significant differences in any limbic lobe structure between the ADHD medication use group and the ADHD non-medication use group (eTables 12-13).

eTable 9. Linear mixed Models Tested: Limbic Lobe Structures and Medication Use in ADHD

Random Effects Models	
RX 1a	
	ROI ~ ICV + Age at baseline + sex + medication status*age + (1 + age |subject)
RX 1b	
	ROI ~ ICV + Age at baseline + sex + medication status*age + (1|subject)
Fixed Effects Models	
Null 0a	
	ROI ~ ICV + age + Age at baseline + sex + (1 + age | subject)
Null 0b	
	ROI ~ ICV + age + Age at baseline + sex + (1|subject)
FX 1a	
	ROI ~ ICV + sex + age + Age at baseline + medication status + (1 + age |subject)
FX 1b	
	ROI ~ ICV + sex + age + Age at baseline + medication status + (1|subject)
FX 2a	
	ROI ~ ICV + sex + age + Age at baseline + medication status * age + (1 + age |subject)
FX 2a	
	ROI ~ ICV + sex + age + Age at baseline + medication status * age + (1|subject)

Note: RX = random effects, FX = fixed effects, ROI = regions of interest, ICV = intracranial volume, age = participant age from baseline (in months). To increase iterability, the variables ICV and age were centre meaned at baseline.

eTable 10: Fit indices of Random Effects: Limbic Lobe Structures and Medication Use in ADHD

**Region of Interest**	**Model**	**AICc**	**BICc**	**Log-likelihood**	**Test**	**LRT (p-value)**
Hippocampus (left)	1a	1600.79	1603.63	-815.11		
	1b	1608.96	1611.78	-819.04	1a vs 1b	**0.016**
Hippocampus (right)	1a	1635.41	1638.24	-833.35		
	1b	1639.63	1642.46	-835.29	1a vs 1b	0.121
Amygdala (left)	1a	1464.67	1467.50	-747.47		
	1b	1467.17	1470.00	-743.62	1a vs 1b	0.286
Amygdala (right)	1a	1447.28	1450.11	-733.50		
	1b	1459.74	1462.57	-739.55	1a vs 1b	**0.001**
Cingulate gyrus (left)	1a	1991.83	1914.66	-980.42		
	1b	1919.54	1922.37	-984.04	1a vs 1b	**0.021**
Cingulate gyrus (right)	1a	1915.19	1918.01	-982.01		
	1b	1917.96	1920.79	-983.20	1a vs 1b	0.249
Orbitofrontal cortex (left)	1a	1989.61	1992.44	-1021.16		
	1b	1989.76	1992.59	-1021.22	1a vs 1b	0.929
Orbitofrontal cortex (right)*****	1a	1989.82	1992.64	-1021.10		
	1b	1990.30	1993.13	-1021.32	1a vs 1b	0.783
Anterior thalamic nuclei (left)*****	1a	983.23	986.06	-486.50		
	1b	983.25	986.07	-486.48	1a vs 1b	0.992
Anterior thalamic nuclei (right)	1a	969.94	972.77	-479.37		
	1b	970.19	973.01	-479.50	1a vs 1b	0.884
Mammillary bodies (left)	1a	883.94	886.77	-433.98		
	1b	885.75	888.57	-434.70	1a vs 1b	0.406
Mammillary bodies (right)	1a	892.22	895.16	-438.02		
	1b	896.54	899.37	-440.43	1a vs 1b	0.121

Note: LRT = likelihood-ratio test. Bold indicates selected random effects model. AIC = Akaike Information Criterion. BIC = Bayesian Information Criterion. *model is singular.

eTable 11: Fit indices of fixed effects: Limbic Lobe Structures and Medication Use in ADHD

Region of Interest	Model	AICc	BICc	Log-likelihood	Test	LRT (p-value)
Hippocampus (left)	0a	1617.35	1620.18			
	1a	1603.08	1505.91	-814.47	0 vs 1	**0.034**
	2a	1599.36	1602.18	-814.31	0 vs 2	0.098
					1 vs 2	0.701
Hippocampus (right)	0b	1652.34	1655.17			
	1b	1640.43	1636.01	-833.73	0 vs 1	**0.034**
	2b	1636.01	1638.84	-833.37	0 vs 2	0.098
					1 vs 2	0.701
Amygdala (left)	0b	1479.64	1482.47			
	1b	1469.21	1472.04	-743.47	0 vs 1	0.192
	2b	1466.83	1469.66	-743.44	0 vs 2	0.417
					1 vs 2	0.827
Amygdala (right)	0a	1449.59	1452.42			
	1a	1440.02	1442.84	-728.28	0 vs 1	0.192
	2a	1436.81	1439.64	-727.92	0 vs 2	0.417
					1 vs 2	0.827
Cingulate gyrus (left)	0a	1931.12	1933.94			
	1a	1918.13	1920.95	-980.47	0 vs 1	0.948
	2a	1911.83	1914.66	-980.42	0 vs 2	0.981
					1 vs 2	0.857
Cingulate gyrus (right)	0b	1936.63	1939.45			
	1b	1923.99	1926.82	-983.30	0 vs 1	0.948
	2b	1918.11	1920.94	-983.28	0 vs 2	0.981
					1 vs 2	0.857
Orbitofrontal cortex (left)	0b	2013.06	2015.88			
	1b	1997.04	1999.87	-1021.35	0 vs 1	0.098
	2b	1989.76	1992.59	-1021.22	0 vs 2	0.225
					1 vs 2	0.617
Orbitofrontal cortex (right)	0b	2017.15	2019.97			
	1b	2001.36	2004.19	-1023.33	0 vs 1	0.131
	2b	1994.05	1996.88	-1023.32	0 vs 2	0.321
					1 vs 2	0.994
Anterior thalamic nuclei (left)	0b	982.62	985.45			
	1b	978.14	980.97	-484.78	0 vs 1	0.896
	2b	979.07	981.90	-484.25	0 vs 2	0.618
					1 vs 2	0.331
Anterior thalamic nuclei (right)	0b	958.15	960.98			
	1b	953.75	956.58	-471.73	0 vs 1	0.749
	2b	954.56	957.39	-471.10	0 vs 2	0.555
					1 vs 2	0.300
Mammillary bodies (left)	0b	887.28	890.11			
	1b	883.08	885.90	-434.75	0 vs 1	0.448
	2b	883.08	885.91	-433.29	0 vs 2	0.209
					1 vs 2	0.110
Mammillary bodies (right)	0b	898.31	901.13			
	1b	893.99	896.81	-440.49	0 vs 1	0.434
	2b	896.54	899.37	-440.43	0 vs 2	0.694
					1 vs 2	0.729

eTable 12: Results of optimal mixed-effects models (without interaction term) analyses: Limbic Lobe Structures and medication use in ADHD

	**ICV**		**Sex**		**Months from baseline**		**Age at baseline**		**Medication Status**	
Region of Interest	***B* (SE)**	**t, p**	***B* (SE)**	**t, p**	***B* (SE)**	**t, p**	***B* (SE)**	**t, p**	***B* (SE)**	**t, p**
Hippocampus (Left)	-1.738e-04 (1.273e-04)	-1.365, 0.176	5.601e+02 (1.295e+02)	4.323, <0.000	6.941e+00 (1.139e+00)	6.092, <0.000	-2.004e+01 (1.020e+02)	-0.197, 0.844	1.175e+02 (5.499e+01)	2.137, 0.035
Hippocampus (Right)	2.150e-04 (1.629e-04)	1.320, 0.190	4.227e+02 (1.302e+02)	3.245, 0.002	7.918e+00 (1.330e+00)	5.953, <0.000	-5.652e+01 (1.036e+02)	-0.546, 0.587	8.538e+01 (7.056e+01)	1.210, 0.228

eTable 13: Results of optimal mixed-effects models (with interaction term) analyses: Limbic Lobe Structures and medication use in ADHD

	**ICV**		**Sex**		**Months from baseline**		**Age at baseline**		**Medication Status**		**Medication Status * Months from baseline**	
Region of Interest	***B* (SE)**	**t, p**	***B* (SE)**	**t, p**	***B* (SE)**	**t, p**	***B* (SE)**	**t, p**	***B* (SE)**	**t, p**	***B* (SE)**	**t, p**
Amygdala (Left)	2.833e-05 (7.736e-05)	0.366, 0.715	2.657e+02 (5.704e+01)	4.657, <0.000	2.287e+00 (8.755e-01)	2.612, 0.011	8.622e+00 (4.550e+01)	0.189, 0850	4.550e+01 (3.677e+01)	1.237, 0.218	-2.692e-01 (1.290e+00)	-0.209, 0.835
Amygdala (Right)	1.434e-04 (7.664e-05)	1.871, 0.064	2.366e+02 (4.811e+01)	4.917, <0.000	2.324e+00 (1.067e+00)	2.178, 0.035	9.393e+00 (3.845e+01)	0.244, 0.808	4.242e+01 (3.359e+01)	1.263, 0.211	-9.010e-01 (1.598e+00)	-0.564, 0.576
Cingulate Gyrus (Left)	3.437e-04 (4.791e-04)	0.717, 0.475	1.410e+03 (5.759e+02)	2.449, 0.017	-8.394e+00 (5.828e+00)	-1.440, 0.157	-2.086e+02, (4.457e+02)	-0.468, 0.641	-1.032e+02 (2.434e+02)	-0.424, 0.673	-2.804e+00 (8.877e+00)	-0.316, 0.753
Cingulate Gyrus (Right)	1.952e-03 (4.906e-04)	3.978, <0.000	5.436e+02 (5.734e+02)	0.948, 0.347	-1.204e+01, (5.058e+00)	-2.380, 0.020	-3.933e+02 (4.447e+02)	-0.884, 0.379	3.312e+00 (2.448e+02)	0.014, 0.989	-1.297e+00 (7.461e+00)	-0.174, 0.862
Orbitofrontal Cortex (Left)	2.269e-03 (8.039e-04)	2.822, 0.005	7.529e+02 (5.746e+02)	1.310, 0.196	-2.965e+01 (9.202e+00)	-3.222, 0.002	3.553e+02 (4.588e+02)	0.774, 0.442	-4.713e+02 (3.805e+02)	-1.238, 0.218	-6.564e+00 (1.356e+01)	-0.484, 0.630
Orbitofrontal Cortex (Right)	2.055e-03 (8.297e-04)	2.477, 0.014	8.571e+02 (4.983e+02)	1.720, 0.091	-3.870e+01 (1.037e+01)	-3.733, <0.000	4.434e+02, (3.999e+02)	1.109, 0.272	-4.927e+02 (3.843e+02)	-1.282, 0.202	-4.849e-02 (1.526e+01)	-0.003, 0.997
Anterior Thalamic Nuclei (Left)	1.146e-05 (9.065e-06)	1.264, 0.209	-2.426e+00 (6.254e+00)	-0.388, 0.700	3.681e-02 (1.053e-01)	0.350, 0.738	5.302e+00 (4.998e+00)	1.061, 0.294	2.341e+00 (4.271e+00)	0.548, 0.585	-1.461e-01 (1.551e-01)	-0.942, 0.350
Anterior Thalamic Nuclei (Right)	2.353e-05 (8.633e-06)	2.726, 0.007	-4.151e+00 (5.681e+00)	-0.731, 0.468	-1.879e-02 (1.024e-01)	-0.184, 0.855	4.598e+00 (4.547e+00)	1.011, 0.316	3.056e+00 (4.042e+00)	0.756, 0.451	-1.520e-01 (1.508e-01)	-1.008, 0.317
Mammillary Bodies (Left)	-4.236e-06 (5.839e-06)	-0.725, 0.469	1.142e+01 (4.110e+00)	2.778, 0.007	4.391e-02 (6.725e-02)	0.653, 0.516	-1.904e+00 (3.283e+00)	-0.580, 0.564	-3.820e+00 (2.758e+00)	-1.385, 0.168	1.567e-01 (9.909e-02)	1.581, 0.118
Mammillary Bodies (Right)	-1.643e-06 (6.151e-06)	-0.267, 0.789	1.046e+01 (4.258e+00)	2.456, 0.017	1.756e-01 (7.132e-02)	2.462, 0.016	-1.903e+00 (3.403e+00)	-0.559, 0.578	1.523e+00 (2.899e+00)	0.525, 0.600	3.605e-02 (1.051e-01)	0.343, 0.732

3. Additional analyses: ADHD-associated brain regions

Additional analyses were performed to examine the between-group differences in brain regions outside the limbic system that have been associated with abnormalities in ADHD, specifically components of the basal ganglia, inferior prefrontal cortex and dorsolateral prefrontal cortex. To measure between-group differences in volume, LME was used via the lme4 package in R (version 1.1-27.1)(2). The chosen LME model is illustrated as model FX2b in eTable 1.

Secondary analyses were also conducted to investigate the relationship between these brain volumes and ADHD symptoms (CAI and ARI) in children and adolescents with ADHD. These relationships were investigated using LME via the lme4 package in R (version 1.1-27.1)(2). The selected LME model, depicted as model FX3b in eTable 1, assessed whether age-related volume changes in these structures varied based on ADHD symptom severity, by including an age-by-ADHD symptoms interaction term. To minimize the impact of multiple comparisons, a two-stage FDR correction was conducted using the MuToss package(3) in R (v.4.1.1).

<u>Inferior Prefrontal Cortex</u>

<u>Left Parsopercularis</u>

eTable 14: Left Parsopercularis in ADHD and Controls.

Fixed effects:	Estimate	Std. Error	t	p
ICV_c	1.679e-04	9.877e-05	1.700	0.090
sex1	1.452e+02	1.281e+02	1.134	0.257
Age_baseline_c	-2.387e+02	1.558e+02	-1.532	0.127
mts_baseline	-3.521e+00	7.557e-01	-4.660	<0.000
diagnosis1	-4.488e+02	1.650e+02	-2.721	0.007
mts_baselinediagnosis1	2.156e+00	1.433e+00	1.504	0.134

eFigure 5. Group difference in left parsopercularis volume.

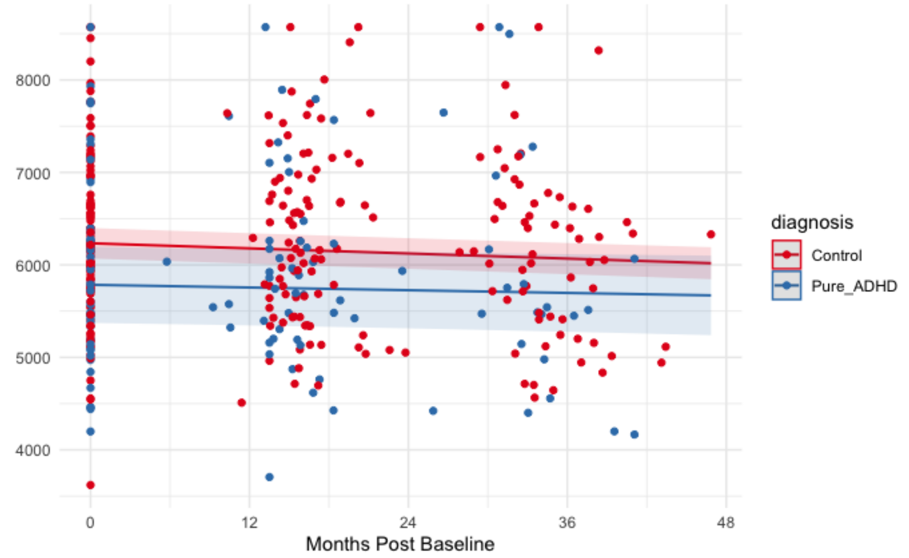

eTable 15: Left Parsopercularis and CAI scores in ADHD.

Fixed effects:	Estimate	Std. Error	t	p
ICV	-9.664e-05	1.585e-04	-0.610	0.544
sex	-2.405e+02	3.278e+02	-0.734	0.466
CAI	3.836e+00	5.993e+00	0.640	0.524
mts_baseline	-5.107e+00	2.979e+00	-1.714	0.092
CAI:mts_baseline	2.193e-01	2.115e-01	1.037	0.304

eTable 16: Left Parsopercularis and ARI scores in ADHD.

Fixed effects:	Estimate	Std. Error	t	p
ICV	-1.815e-04	3.131e-04	-0.580	0.565
Sex	-1.773e+02	3.359e+02	-0.528	0.600
ARI	-1.299e+01	1.245e+01	-1.043	0.303
mts_baseline	-9.989e-01	3.036e+00	-0.329	0.744
ARI:mts_baseline	3.348e-01	5.281e-01	0.634	0.530

Right Parsopercularis

eTable 17: Right Parsopercularis in ADHD and Controls.

Fixed effects:	Estimate	Std. Error	t	p
ICV_c	6.401e-04	1.253e-04	5.109	<0.000
sex1	9.052e+01	1.138e+02	0.796	0.427
Age_baseline_c	-7.135e+01	1.246e+02	-0.573	0.568
mts_baseline	-5.862e+00	9.789e-01	-5.989	<0.000
diagnosis1	-4.701e+01	1.278e+02	-0.368	0.714
mts_baselinediagnosis1	-1.663e+00	1.861e+00	-0.894	0.373

eFigure 6. Group difference in right parsopercularis volume.

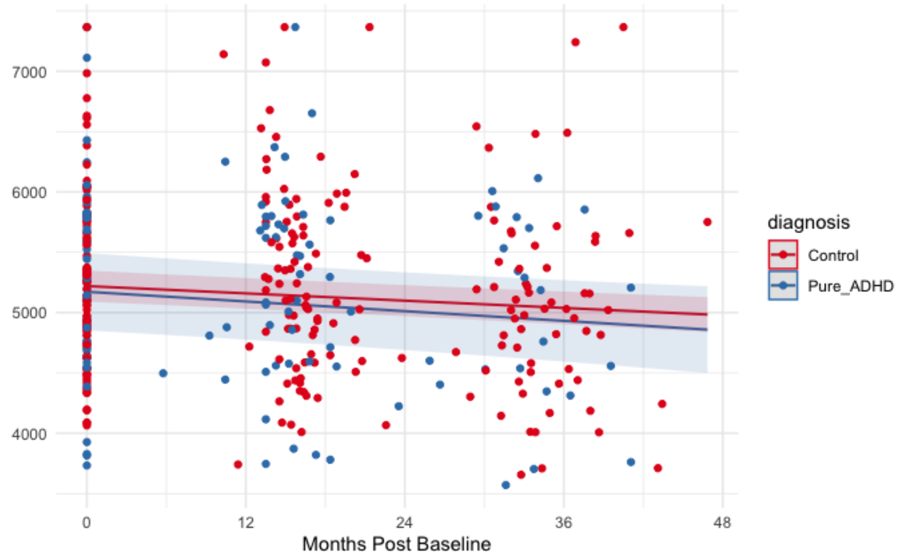

eTable 18: Right Parsopercularis and CAI scores in ADHD.

Fixed effects:	Estimate	Std. Error	t	p
ICV	9.924e-04	2.671e-04	3.716	<0.000
sex	7.194e+01	2.522e+02	0.285	0.776
CAI	2.107e+00	1.008e+01	0.209	0.835
mts_baseline	5.294e-01	5.335e+00	0.099	0.921
CAI:mts_baseline	-5.477e-01	3.799e-01	-1.442	0.154

eTable 19: Right Parsopercularis and ARI scores in ADHD.

Fixed effects:	Estimate	Std. Error	t	p
ICV	2.415e-03	4.162e-04	5.803	<0.000
Sex	-1.102e+02	2.562e+02	-0.430	0.668
ARI	1.488e+01	1.721e+01	0.865	0.391
mts_baseline	2.279e+00	4.543e+00	0.502	0.619
ARI:mts_baseline	-1.666e+00	7.771e-01	-2.144	0.038

<u>Left Parsorbitalis</u>

eTable 20: Left parsorbitalis in ADHD and Controls.

Fixed effects:	Estimate	Std. Error	t	p
ICV_c	1.790e-04	8.469e-05	2.113	0.035
sex1	9.671e+01	6.584e+01	1.469	0.143
Age_baseline_c	-1.205e+01	7.027e+01	-0.172	0.864
mts_baseline	-3.497e+00	6.725e-01	-5.201	<0.000
diagnosis1	-6.704e+00	7.210e+01	-0.093	0.926
mts_baselinediagnosis1	1.245e+00	1.281e+00	0.972	0.332

eFigure 7. Group difference in left parsorbitalis volume.

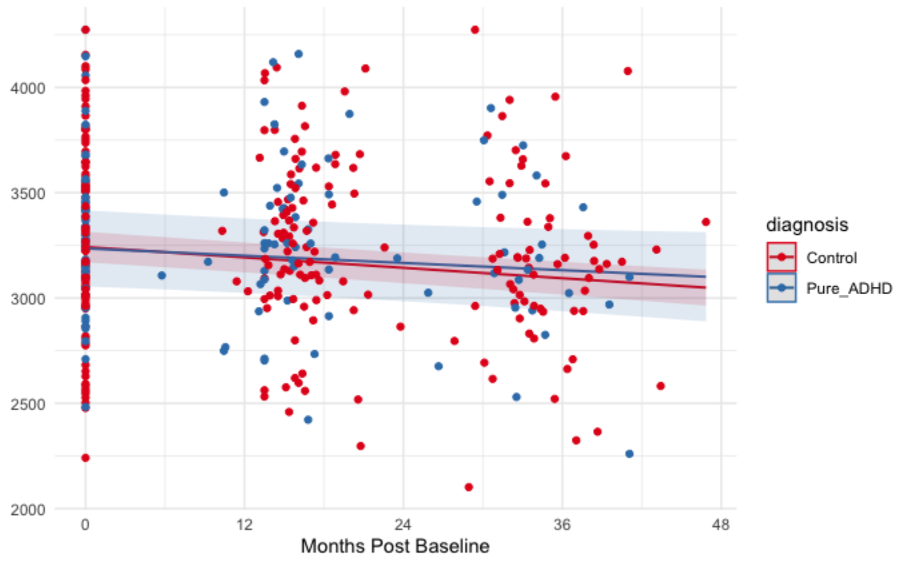

eTable 21: Left parsorbitalis and CAI scores in ADHD.

Fixed effects:	Estimate	Std. Error	t	p
ICV	6.617e-05	1.195e-04	0.554	0.582
sex	9.070e+01	1.259e+02	0.720	0.475
CAI	-1.484e+00	4.514e+00	-0.329	0.743
mts_baseline	-5.202e+00	2.349e+00	-2.214	0.031
CAI:mts_baseline	1.247e-01	1.672e-01	0.746	0.459

eTable 22: Left parsorbitalis and ARI scores in ADHD.

Fixed effects:	Estimate	Std. Error	t	p
ICV	3.788e-04	1.724e-04	2.197	0.032
Sex	5.258e+01	1.167e+02	0.451	0.654
ARI	-8.908e+00	7.051e+00	-1.263	0.212
mts_baseline	-1.516e+00	1.818e+00	-0.834	0.410
ARI:mts_baseline	-1.201e-01	3.125e-01	-0.384	0.703

Right Parsorbitalis

eTable 23: Right parsorbitalis in ADHD and Controls.

Fixed effects:	Estimate	Std. Error	t	p
ICV_c	4.544e-04	1.426e-04	3.187	0.001
sex1	2.196e+02	8.226e+01	2.670	0.008
Age_baseline_c	-1.862e+01	8.479e+01	-0.220	0.826
mts_baseline	-6.699e+00	1.197e+00	-5.599	<0.000
diagnosis1	-1.599e+02	8.860e+01	-1.804	0.072
mts_baselinediagnosis1	1.367e+00	2.292e+00	0.596	0.551

eFigure 8. Group difference in right parsorbitalis volume.

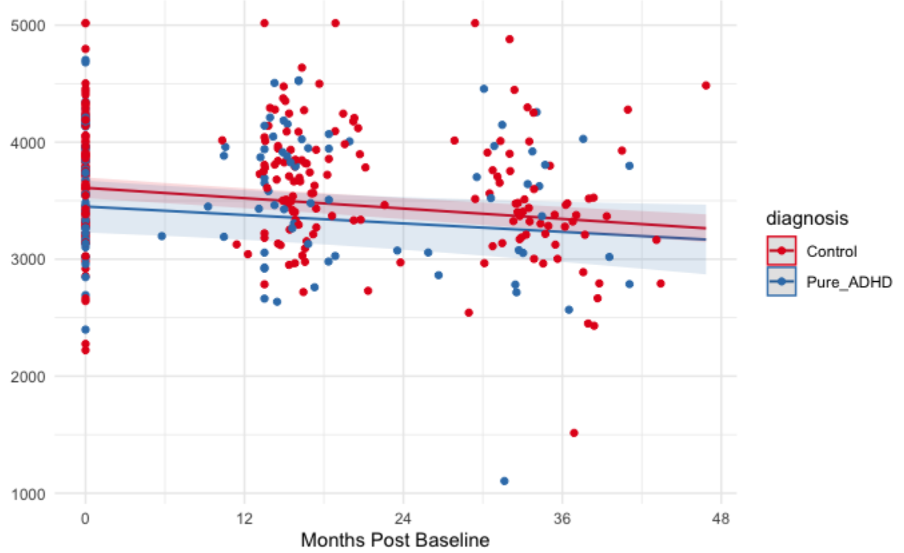

eTable 24: Right parsorbitalis and CAI scores in ADHD.

Fixed effects:	Estimate	Std. Error	t	p
ICV	5.089e-04	2.280e-04	2.233	0.028
sex	2.185e+02	1.658e+02	1.318	0.193
CAI	-1.448e+01	8.621e+00	-1.679	0.096
mts_baseline	-1.115e+01	4.855e+00	-2.297	0.025
CAI:mts_baseline	3.030e-01	3.465e-01	0.875	0.385

eTable 25: Right parsorbitalis and ARI scores in ADHD.

Fixed effects:	Estimate	Std. Error	t	p
ICV	1.623e-03	2.997e-04	5.415	<0.000
Sex	3.592e+00	1.470e+02	0.024	0.981
ARI	-1.578e+01	1.293e+01	-1.220	0.227
mts_baseline	-1.757e+00	3.756e+00	-0.468	0.643
ARI:mts_baseline	-6.236e-01	6.312e-01	-0.988	0.329

Left Parstriangularis

eTable 26: Left parstriangularis in ADHD and Controls.

Fixed effects:	Estimate	Std. Error	t	p
ICV_c	2.629e-04	9.900e-05	2.656	0.008
sex1	2.213e+02	1.097e+02	2.018	0.044
Age_baseline_c	3.286e+01	1.259e+02	0.261	0.794
mts_baseline	-3.133e+00	7.630e-01	-4.106	<0.000
diagnosis1	-7.908e+01	1.305e+02	-0.606	0.545
mts_baselinediagnosis1	8.370e-01	1.448e+00	0.578	0.563

eFigure 9. Group difference in left parstriangularis volume.

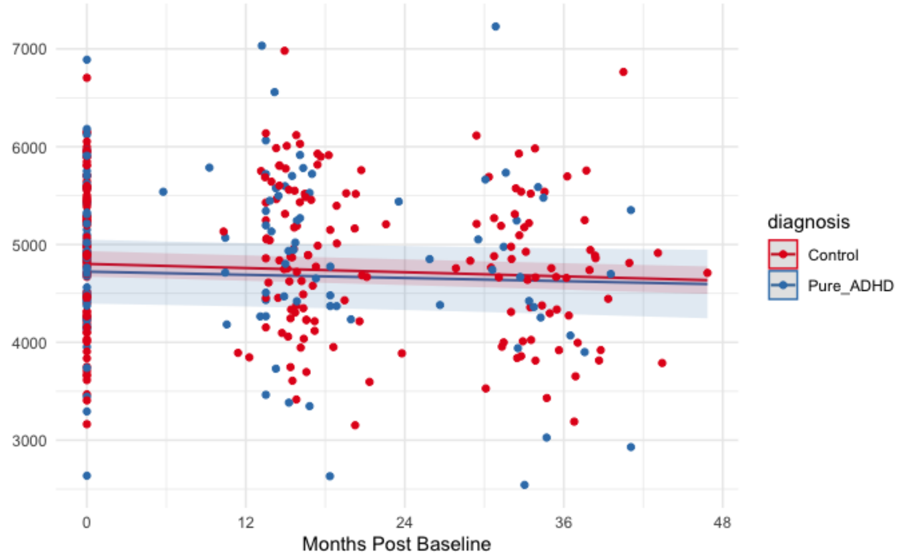

eTable 27: Left parstriangularis and CAI scores in ADHD.

Fixed effects:	Estimate	Std. Error	t	p
ICV	1.371e-05	1.471e-04	0.093	0.926
sex	5.104e+02	2.658e+02	1.920	0.060
CAI	-3.459e-01	5.560e+00	-0.062	0.950
mts_baseline	-6.152e+00	2.777e+00	-2.215	0.031
CAI:mts_baseline	2.359e-01	1.972e-01	1.196	0.237

eTable 28: Left parstriangularis and ARI scores in ADHD.

Fixed effects:	Estimate	Std. Error	t	p
ICV	1.831e-04	2.220e-04	0.824	0.414
Sex	5.005e+02	2.656e+02	1.884	0.065
ARI	-1.335e+01	8.799e+00	-1.517	0.137
mts_baseline	-1.047e+00	2.132e+00	-0.491	0.626
ARI:mts_baseline	5.683e-01	3.714e-01	1.530	0.135

Right Parstriangularis

eTable 29: Right parstriangularis in ADHD and Controls.

Fixed effects:	Estimate	Std. Error	t	p
ICV_c	7.871e-04	1.976e-04	3.983	<0.000
sex1	5.401e+02	1.451e+02	3.722	<0.000
Age_baseline_c	-1.133e+02	1.537e+02	-0.737	0.461
mts_baseline	-1.181e+01	1.581e+00	-7.469	<0.000
diagnosis1	-2.930e+02	1.579e+02	-1.855	0.0652
mts_baselinediagnosis1	-4.966e-01	3.014e+00	-0.165	0.869

eFigure 10. Group difference in right parstriangularis volume.

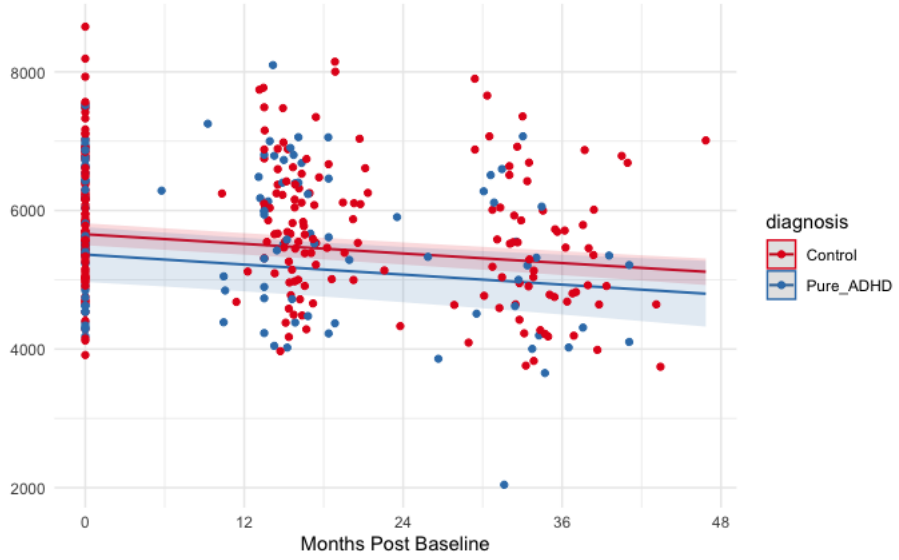

eTable 30: Right parstriangularis and CAI scores in ADHD.

Fixed effects:	Estimate	Std. Error	t	p
ICV	1.138e-03	3.930e-04	2.895	0.004
sex	9.268e+02	2.937e+02	3.155	0.002
CAI	-2.027e+01	1.486e+01	-1.365	0.175
mts_baseline	-7.566e+00	8.293e+00	-0.912	0.365
CAI:mts_baseline	-4.330e-01	5.917e-01	-0.732	0.467

eTable 31: Right parstriangularis and ARI scores in ADHD.

Fixed effects:	Estimate	Std. Error	t	p
ICV	3.025e-03	5.925e-04	5.105	<0.000
Sex	5.857e+02	3.073e+02	1.906	0.062
ARI	1.141e+01	2.523e+01	0.452	0.652
mts_baseline	7.799e+00	7.101e+00	1.098	0.279
ARI:mts_baseline	-3.097e+00	1.200e+00	-2.581	0.013

Dorsolateral Prefrontal Cortex

Left Caudal Middle Frontal

eTable 32: Left caudal middle frontal in ADHD and Controls.

Fixed effects:	Estimate	Std. Error	t	p
ICV_c	2.382e-04	1.422e-04	1.675	0.095
sex1	4.319e+02	1.775e+02	2.433	0.015
Age_baseline_c	-3.353e+02	2.124e+02	-1.578	0.116
mts_baseline	-5.784e+00	1.089e+00	-5.310	<0.000
diagnosis1	-7.539e+02	2.235e+02	-3.374	<0.000
mts_baselinediagnosis1	1.388e+00	2.067e+00	0.671	0.502

eFigure 11. Group difference in left caudal middle frontal volume.

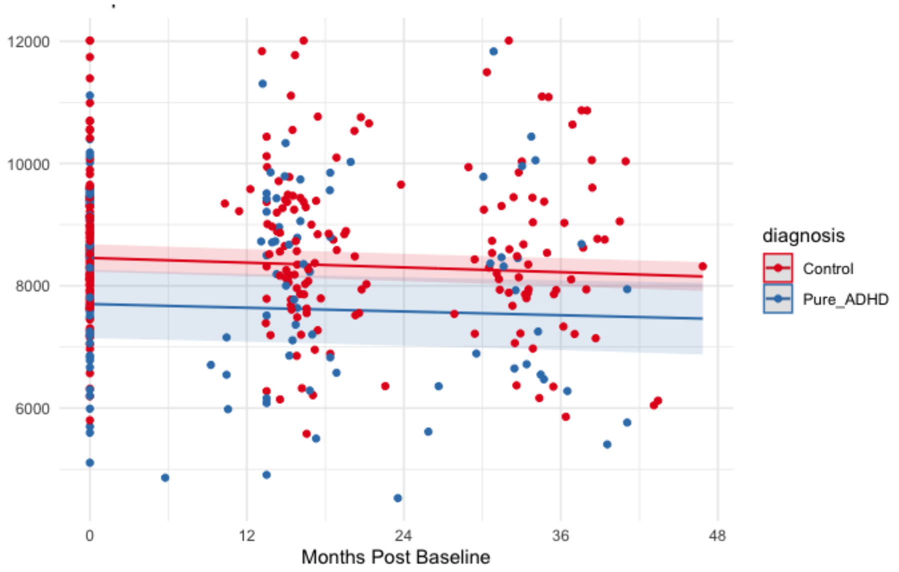

eTable 33: Left caudal middle frontal and CAI scores in ADHD.

Fixed effects:	Estimate	Std. Error	t	p
ICV	6.489e+02	4.778e+02	1.358	0.181
sex	-6.259e+00	9.752e+00	-0.642	0.524
CAI	-7.531e+00	4.866e+00	-1.548	0.128
mts_baseline	3.300e-02	3.456e-01	0.095	0.924
CAI:mts_baseline	6.489e+02	4.778e+02	1.358	0.181

eTable 34: Left caudal middle frontal and ARI scores in ADHD.

Fixed effects:	Estimate	Std. Error	t	p
ICV	1.111e-03	3.540e-04	3.138	0.003
Sex	5.699e+02	4.426e+02	1.287	0.204
ARI	-2.342e+01	1.401e+01	-1.672	0.102
mts_baseline	-4.345e+00	3.388e+00	-1.283	0.208
ARI:mts_baseline	-1.258e-01	5.904e-01	-0.213	0.832

Right Caudal Middle Frontal

eTable 35: Right caudal middle frontal in ADHD and Controls.

Fixed effects:	Estimate	Std. Error	t	p
ICV_c	7.197e-04	1.801e-04	3.996	<0.000
sex1	2.751e+02	1.960e+02	1.403	0.161
Age_baseline_c	-1.806e+02	2.239e+02	-0.807	0.420
mts_baseline	-8.114e+00	1.389e+00	-5.840	<0.000
diagnosis1	-4.553e+02	2.318e+02	-1.964	0.051
mts_baselinediagnosis1	1.094e+00	2.638e+00	0.415	0.678

eFigure 12. Group difference in right caudal middle frontal volume.

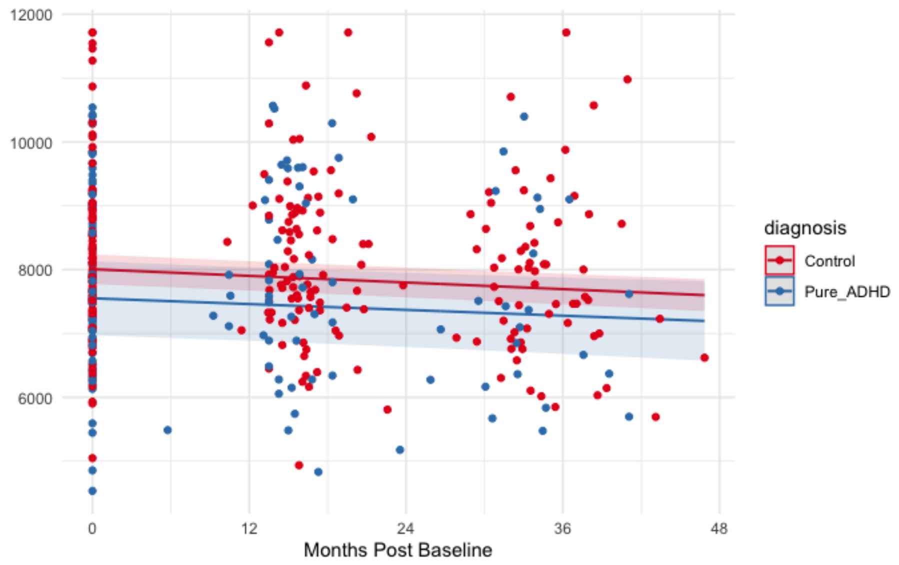

eTable 36: Right caudal middle frontal and CAI scores in ADHD.

Fixed effects:	Estimate	Std. Error	t	p
ICV	8.044e-04	3.413e-04	2.357	0.021
sex	-3.914e+01	4.942e+02	-0.079	0.937
CAI	-8.209e+00	1.289e+01	-0.637	0.526
mts_baseline	-1.870e+00	6.514e+00	-0.287	0.775
CAI:mts_baseline	-5.716e-01	4.629e-01	-1.235	0.222

eTable 37: Right caudal middle frontal and ARI scores in ADHD.

Fixed effects:	Estimate	Std. Error	t	p
ICV	2.805e-03	5.271e-04	5.32	<0.000
Sex	-2.477e+02	4.831e+02	-0.513	0.611
ARI	2.398e+00	2.109e+01	0.114	0.910
mts_baseline	-1.188e+00	5.209e+00	-0.228	0.821
ARI:mts_baseline	-1.340e+00	9.035e-01	-1.483	0.147

Left Rostral Middle Frontal

eTable 38: Left rostral middle frontal in ADHD and Controls.

Fixed effects:	Estimate	Std. Error	t	p
ICV_c	2.389e-03	4.729e-04	5.052	<0.000
sex1	1.567e+03	3.943e+02	3.973	<0.000
Age_baseline_c	-4.865e+02	4.254e+02	-1.144	0.254
mts_baseline	-3.971e+01	3.725e+00	-10.662	<0.000
diagnosis1	-1.524e+02	4.362e+02	-0.349	0.727
mts_baselinediagnosis1	3.772e+00	7.088e+00	0.532	0.595

eFigure 13. Group difference in left rostral middle frontal volume.

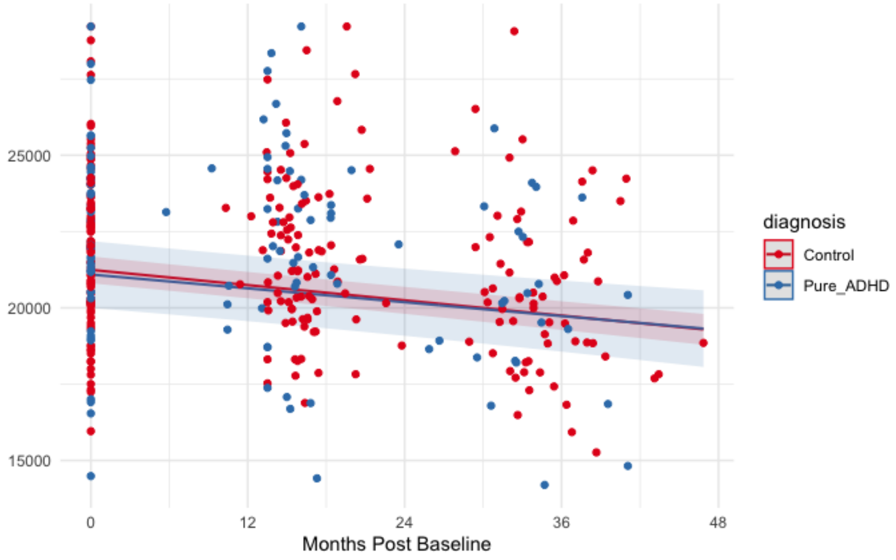

eTable 39: Left rostral middle frontal and CAI scores in ADHD.

Fixed effects:	Estimate	Std. Error	t	p
ICV	1.785e-03	8.232e-04	2.169	0.033
sex	1.578e+03	9.595e+02	1.645	0.106
CAI	-8.133e+00	3.109e+01	-0.262	0.794
mts_baseline	-4.499e+01	1.599e+01	-2.813	0.006
CAI:mts_baseline	2.627e-01	1.137e+00	0.231	0.818

eTable 40: Left rostral middle frontal and ARI scores in ADHD.

Fixed effects:	Estimate	Std. Error	t	p
ICV	1.195e-02	1.465e-03	8.157	<0.000
Sex	4.788e+02	8.084e+02	0.592	0.556
ARI	-1.033e+02	6.159e+01	-1.678	0.098
mts_baseline	-5.619e+00	1.686e+01	-0.333	0.740
ARI:mts_baseline	-6.061e+00	2.864e+00	-2.116	0.040

Right Rostral Middle Frontal

eTable _41 Right rostral middle frontal in ADHD and Controls.

Fixed effects:	Estimate	Std. Error	t	p
ICV_c	4.614e-03	7.828e-04	5.894	<0.000
sex1	1.311e+03	4.382e+02	2.992	0.003
Age_baseline_c	-7.212e+02	4.503e+02	-1.601	0.111
mts_baseline	-7.835e+01	6.625e+00	-11.825	<0.000
diagnosis1	-3.033e+02	4.723e+02	-0.642	0.521
mts_baselinediagnosis1	5.367e+00	1.270e+01	0.423	0.673

eFigure 14. Group difference in right rostral middle frontal volume.

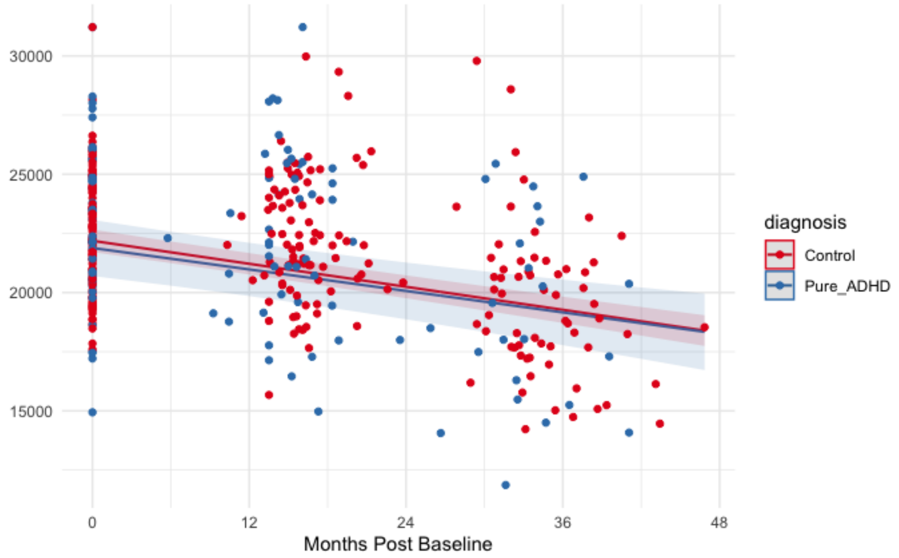

eTable 42: Right rostral middle frontal and CAI scores in ADHD.

Fixed effects:	Estimate	Std. Error	t	p
ICV	5.150e-03	1.355e-03	3.802	<0.000
sex	1.702e+03	1.004e+03	1.696	0.095
CAI	-7.182e+01	5.121e+01	-1.402	0.164
mts_baseline	-7.483e+01	2.866e+01	-2.611	0.011
CAI:mts_baseline	-4.049e-01	2.045e+00	-0.198	0.843

eTable 43: Right rostral middle frontal and ARI scores in ADHD.

Fixed effects:	Estimate	Std. Error	t	p
ICV	3.886e-03	1.007e-03	3.860	<0.000
Sex	1.363e+03	9.068e+02	1.503	0.139
ARI	-3.781e+01	4.033e+01	-0.937	0.354
mts_baseline	-7.606e+00	9.977e+00	-0.762	0.451
ARI:mts_baseline	-4.859e-01	1.730e+00	-0.281	0.780

Thalamus

Left Thalamus

eTable 44: Left thalamus in ADHD and Controls.

Fixed effects:	Estimate	Std. Error	t	p
ICV_c	4.755e-04	1.036e-04	4.589	<0.000
sex1	4.596e+02	9.883e+01	4.650	<0.000
Age_baseline_c	-2.673e+01	1.093e+02	-0.245	0.807
mts_baseline	1.325e+00	8.064e-01	1.643	0.101
diagnosis1	-2.520e+02	1.123e+02	-2.244	0.026
mts_baselinediagnosis1	-1.911e+00	1.533e+00	-1.247	0.213

eFigure 15. Group difference in left thalamus volume.

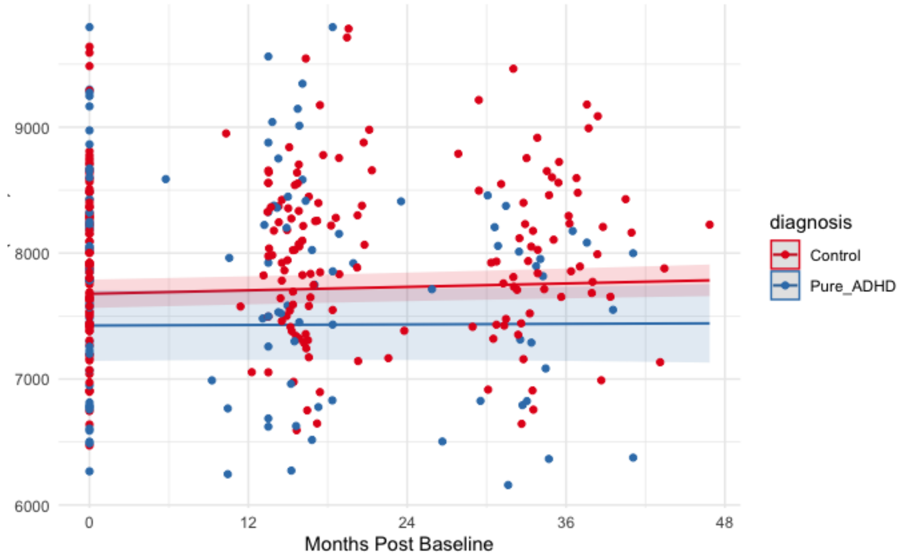

eTable 45: Left thalamus and CAI scores in ADHD.

Fixed effects:	Estimate	Std. Error	t	p
ICV	5.674e-04	2.354e-04	2.411	0.018
sex	8.944e+02	2.481e+02	3.605	<0.000
CAI	-1.799e+01	8.888e+00	-2.024	0.046
mts_baseline	5.117e-01	4.625e+00	0.111	0.912
CAI:mts_baseline	-2.028e-02	3.291e-01	-0.062	0.951

eTable 46: Left thalamus and ARI scores in ADHD.

Fixed effects:	Estimate	Std. Error	t	p
ICV	1.887e-03	3.993e-04	4.725	<0.000
Sex	6.627e+02	2.274e+02	2.914	0.005
ARI	-1.031e+01	1.670e+01	-0.617	0.539
mts_baseline	4.631e+00	4.518e+00	1.025	0.312
ARI:mts_baseline	-1.024e+00	7.692e-01	-1.331	0.191

Right Thalamus

eTable 47: Right thalamus in ADHD and Controls.

Fixed effects:	Estimate	Std. Error	t	p
ICV_c	5.281e-04	1.119e-04	4.720	<0.000
sex1	3.802e+02	9.395e+01	4.047	<0.000
Age_baseline_c	6.315e+00	1.015e+02	0.062	0.950
mts_baseline	2.098e+00	8.807e-01	2.382	0.018
diagnosis1	-1.656e+02	1.040e+02	-1.592	0.113
mts_baselinediagnosis1	-2.995e+00	1.676e+00	-1.787	0.075

eFigure 16. Group difference in right thalamus volume.

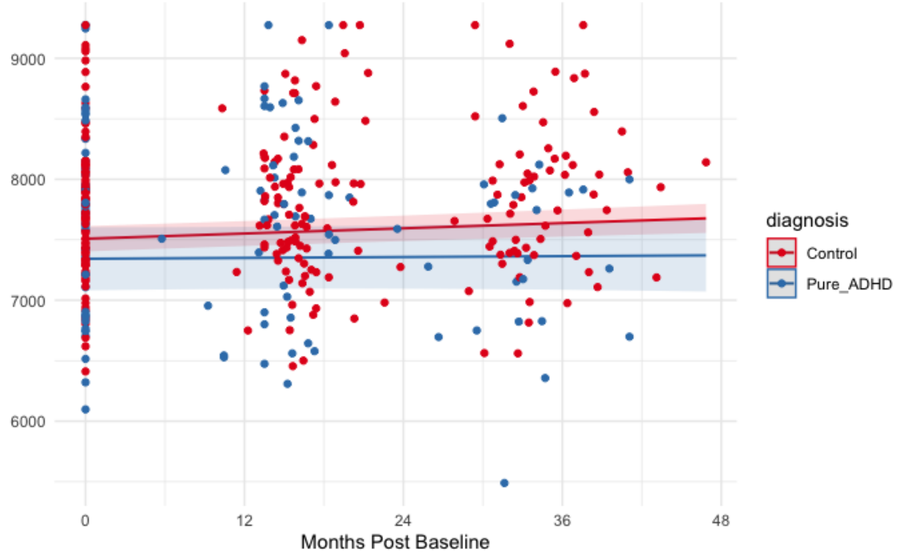

eTable 48: Right thalamus and CAI scores in ADHD.

Fixed effects:	Estimate	Std. Error	t	p
ICV	8.550e-04	2.786e-04	3.069	0.002
sex	6.528e+02	2.153e+02	3.032	0.003
cp_index	-1.948e+01	1.053e+01	-1.851	0.067
mts_baseline	3.548e+00	5.820e+00	0.610	0.544
cp_index:mts_baseline	-2.796e-01	4.152e-01	-0.674	0.503

eTable 49: Right thalamus and ARI scores in ADHD.

Fixed effects:	Estimate	Std. Error	t	p
ICV	2.666e-03	4.184e-04	6.372	<0.000
Sex	3.622e+02	1.836e+02	1.973	0.054
ARI	-1.855e+01	1.874e+01	-0.990	0.325
mts_baseline	2.094e+00	5.974e+00	0.351	0.727
ARI:mts_baseline	-7.587e-01	9.884e-01	-0.768	0.446

Basal Ganglia

Left Caudate Nucleus

eTable 50: Left caudate nucleus in ADHD and Controls.

Fixed effects:	Estimate	Std. Error	t	p
ICV_c	2.625e-04	5.918e-05	4.436	<0.000
sex1	1.047e+02	6.999e+01	1.495	0.136
Age_baseline_c	-1.092e+02	8.206e+01	-1.330	0.185
mts_baseline	-3.248e+00	4.546e-01	-7.144	<0.000
diagnosis1	-9.252e+01	8.567e+01	-1.080	0.281
mts_baselinediagnosis1	-2.050e+00	8.627e-01	-2.376	0.018

eFigure 17. Group difference in left caudate nucleus volume.

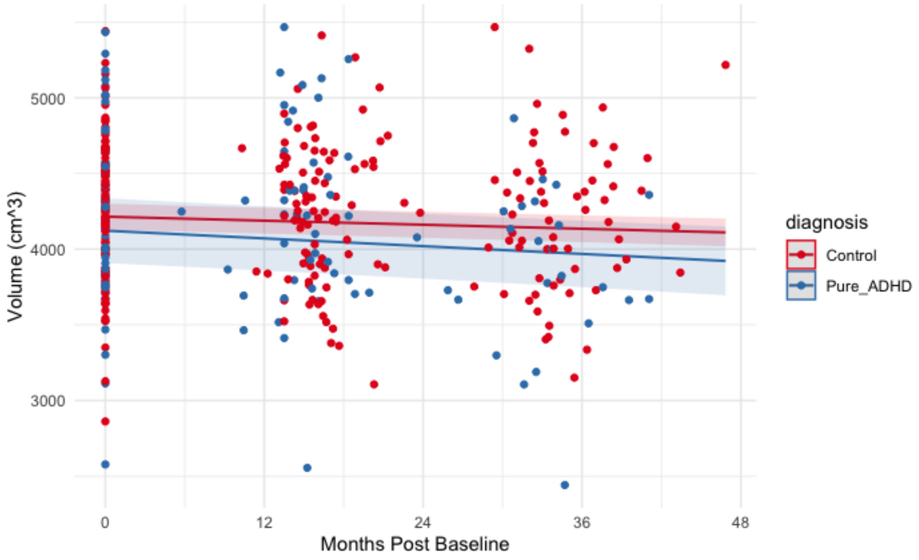

eTable 51: Left caudate nucleus and CAI scores in ADHD.

Fixed effects:	Estimate	Std. Error	t	p
ICV	3.636e-04	1.458e-04	2.494	0.015
sex	4.371e+02	1.763e+02	2.480	0.016
CAI	-1.130e+01	5.507e+00	-2.052	0.044
mts_baseline	-3.013e+00	2.822e+00	-1.068	0.290
CAI:mts_baseline	-1.237e-01	2.007e-01	-0.616	0.540

eTable 52: Left caudate nucleus and ARI scores in ADHD.

Fixed effects:	Estimate	Std. Error	t	p
ICV	1.438e-03	2.342e-04	6.142	<0.000
Sex	2.884e+02	1.554e+02	1.857	0.069
ARI	-8.277e+00	9.598e+00	-0.862	0.392
mts_baseline	-1.028e+00	2.486e+00	-0.414	0.681
ARI:mts_baseline	-4.501e-01	4.269e-01	-1.054	0.298

Right Caudate Nucleus

eTable 53: Right caudate nucleus in ADHD and Controls.

Fixed effects:	Estimate	Std. Error	t	p
ICV_c	5.090e-04	9.014e-05	5.646	<0.000
sex1	1.205e+02	7.595e+01	1.587	0.114
Age_baseline_c	-4.851e+01	8.206e+01	-0.591	0.555
mts_baseline	-2.834e+00	7.093e-01	-3.996	<0.000
diagnosis1	-7.644e+01	8.415e+01	-0.908	0.364
mts_baselinediagnosis1	-3.558e+00	1.350e+00	-2.636	<0.000

eFigure 18. Group difference in right caudate nucleus volume.

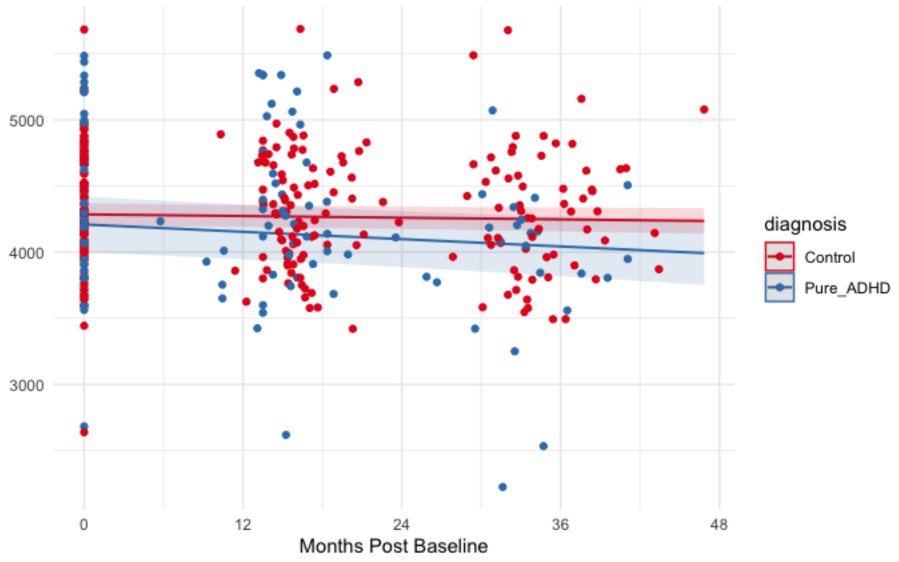

eTable 54: Right caudate nucleus and CAI scores in ADHD.

Fixed effects:	Estimate	Std. Error	t	p
ICV	9.247e-04	2.391e-04	3.867	<0.000
sex	3.977e+02	1.776e+02	2.239	0.029
CAI	-1.704e+01	9.040e+00	-1.885	0.062
mts_baseline	-7.663e-01	5.056e+00	-0.152	0.880
CAI:mts_baseline	-3.664e-01	3.608e-01	-1.016	0.314

eTable 55: Right caudate nucleus and ARI scores in ADHD.

Fixed effects:	Estimate	Std. Error	t	p
ICV	2.485e-03	3.391e-04	7.329	<0.000
Sex	1.464e+02	1.490e+02	0.983	0.330
ARI	-1.534e+01	1.518e+01	-1.010	0.316
mts_baseline	-1.167e+00	4.833e+00	-0.241	0.810
ARI:mts_baseline	-1.069e+00	7.998e-01	-1.337	0.188

Left Putamen

eTable 56: Left putamen nucleus in ADHD and Controls.

Fixed effects:	Estimate	Std. Error	t	p
ICV_c	1.911e-04	8.966e-05	2.132	0.034
sex1	3.752e+02	8.307e+01	4.516	<0.000
Age_baseline_c	-9.530e+01	9.131e+01	-1.044	0.298
mts_baseline	7.536e-01	6.994e-01	1.078	0.282
diagnosis1	-1.200e+02	9.375e+01	-1.280	0.202
mts_baselinediagnosis1	-1.187e+00	1.329e+00	-0.893	0.372

eFigure 19. Group difference in left putamen volume.

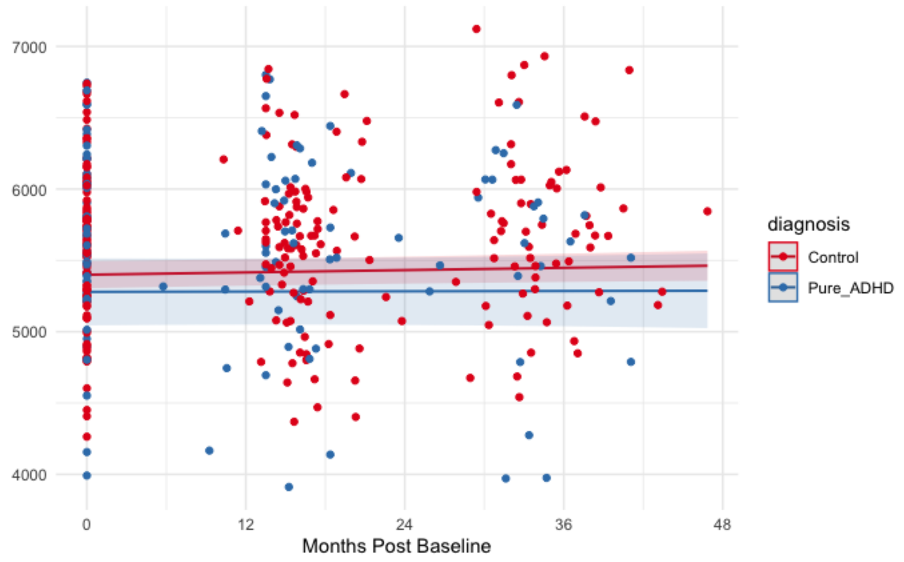

eTable 57: Left putamen and CAI scores in ADHD.

Fixed effects:	Estimate	Std. Error	t	p
ICV	4.405e-04	1.953e-04	2.255	0.027
sex	5.346e+02	1.899e+02	2.816	0.006
CAI	-1.452e+01	7.375e+00	-1.969	0.052
mts_baseline	4.928e-02	3.883e+00	0.013	0.989
CAI:mts_baseline	-1.622e-02	2.765e-01	-0.059	0.953

eTable 58: Left putamen and ARI scores in ADHD.

Fixed effects:	Estimate	Std. Error	t	p
ICV	1.602e-03	3.399e-04	4.713	<0.000
Sex	3.282e+02	1.722e+02	1.906	0.062
ARI	-1.662e+01	1.455e+01	-1.143	0.257
mts_baseline	-5.525e+00	4.146e+00	-1.333	0.191
ARI:mts_baseline	2.732e-01	6.991e-01	0.391	0.698

Right Putamen

eTable 59: Right putamen nucleus in ADHD and Controls.

Fixed effects:	Estimate	Std. Error	t	p
ICV_c	4.371e-05	7.523e-05	0.581	0.561
sex1	4.036e+02	7.818e+01	5.162	<0.000
Age_baseline_c	-7.113e+01	8.819e+01	-0.807	0.421
mts_baseline	2.200e+00	5.820e-01	3.780	<0.000
diagnosis1	-8.884e+01	9.100e+01	-0.976	0.330
mts_baselinediagnosis1	-9.466e-02	1.105e+00	-0.086	0.931

eFigure 20. Group difference in right putamen volume.

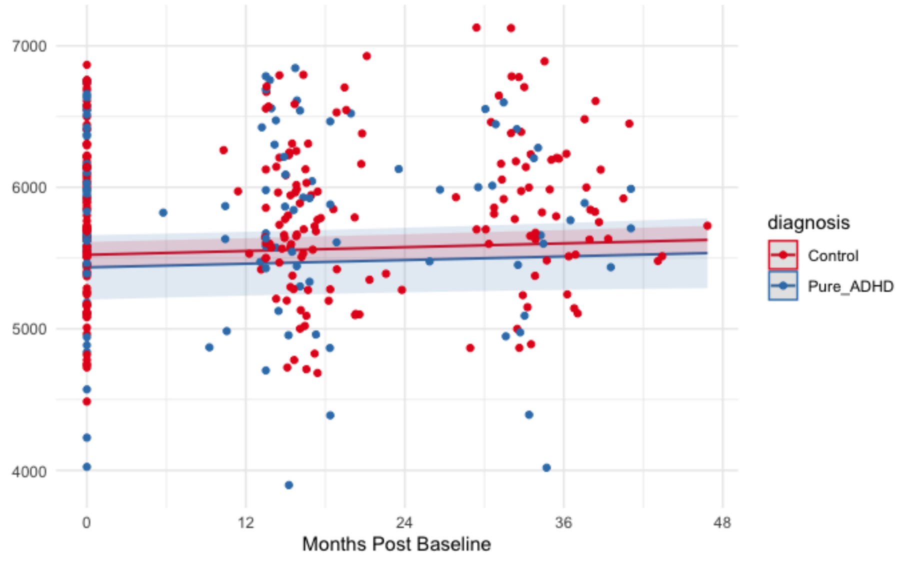

eTable 60: Right putamen and CAI scores in ADHD.

Fixed effects:	Estimate	Std. Error	t	p
ICV	6.259e-05	1.539e-04	0.407	0.685
sex	6.098e+02	1.924e+02	3.169	0.002
CAI	-7.415e+00	5.813e+00	-1.276	0.206
mts_baseline	9.978e-01	2.970e+00	0.336	0.738
CAI:mts_baseline	7.926e-02	2.111e-01	0.375	0.708

eTable 61: Right putamen and ARI scores in ADHD.

Fixed effects:	Estimate	Std. Error	t	p
ICV	7.772e-04	2.764e-04	2.812	0.006
Sex	4.944e+02	1.796e+02	2.752	0.008
ARI	-1.124e+01	1.136e+01	-0.990	0.327
mts_baseline	-2.729e+00	2.956e+00	-0.923	0.363
ARI:mts_baseline	2.120e-01	5.071e-01	0.418	0.678

Left Globus Pallidus

eTable 62: Left globus pallidus nucleus in ADHD and Controls.

Fixed effects:	Estimate	Std. Error	t	p
ICV_c	2.303e-04	4.547e-05	5.064	<0.000
sex1	8.431e+01	3.187e+01	2.645	0.008
Age_baseline_c	1.674e+01	3.356e+01	0.499	0.618
mts_baseline	1.449e+00	3.664e-01	3.954	<0.000
diagnosis1	-1.525e+01	3.454e+01	-0.442	0.659
mts_baselinediagnosis1	-9.317e-01	6.989e-01	-1.333	0.184

eFigure 21. Group difference in left globus pallidus volume.

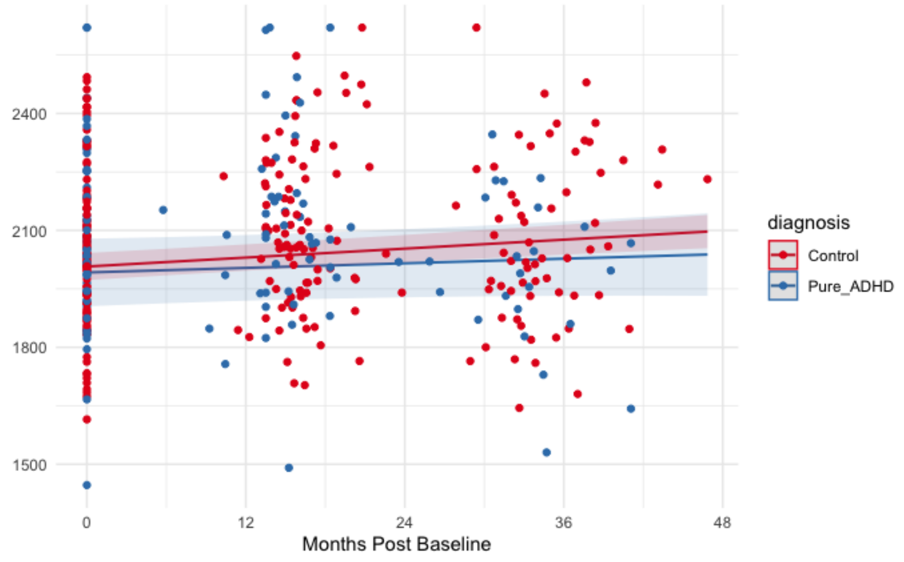

eTable 63: Left globus pallidus and CAI scores in ADHD.

Fixed effects:	Estimate	Std. Error	t	p
ICV	4.405e-04	1.953e-04	2.255	0.027
sex	5.346e+02	1.899e+02	2.816	0.006
CAI	-1.452e+01	7.375e+00	-1.969	0.052
mts_baseline	4.928e-02	3.883e+00	0.013	0.989
CAI:mts_baseline	-1.622e-02	2.765e-01	-0.059	0.953

eTable 64: Left globus pallidus and ARI scores in ADHD.

Fixed effects:	Estimate	Std. Error	t	p
ICV	1.602e-03	3.399e-04	4.713	<0.000
Sex	3.282e+02	1.722e+02	1.906	0.062
ARI	-1.662e+01	1.455e+01	-1.143	0.257
mts_baseline	-5.525e+00	4.146e+00	-1.333	0.191
ARI:mts_baseline	2.732e-01	6.991e-01	0.391	0.698

Right Globus Pallidus

eTable 65: Right globus pallidus nucleus in ADHD and Controls.

Fixed effects:	Estimate	Std. Error	t	p
ICV_c	1.548e-04	4.241e-05	3.650	<0.000
sex1	6.947e+01	3.184e+01	2.182	0.030
Age_baseline_c	2.485e+00	3.382e+01	0.073	0.941
mts_baseline	1.039e+00	3.383e-01	3.072	0.002
diagnosis1	-1.127e+01	3.473e+01	-0.325	0.745
mts_baselinediagnosis1	-4.589e-01	6.447e-01	-0.712	0.477

eFigure 22. Group difference in right globus pallidus volume.

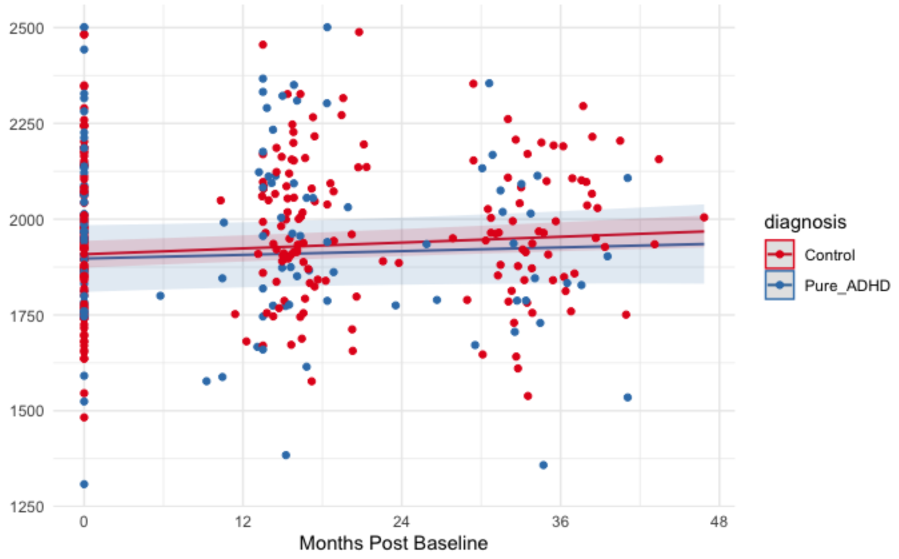

eTable 66: Right globus pallidus and CAI scores in ADHD.

Fixed effects:	Estimate	Std. Error	t	p
ICV	6.259e-05	1.539e-04	0.407	0.685
sex	6.098e+02	1.924e+02	3.169	0.002
CAI	-7.415e+00	5.813e+00	-1.276	0.206
mts_baseline	9.978e-01	2.970e+00	0.336	0.738
CAI:mts_baseline	7.926e-02	2.111e-01	0.375	0.708

eTable 67: Right globus pallidus and ARI scores in ADHD.

Fixed effects:	Estimate	Std. Error	t	p
ICV	7.772e-04	2.764e-04	2.812	0.006
Sex	4.944e+02	1.796e+02	2.752	0.008
ARI	-1.124e+01	1.136e+01	-0.990	0.327
mts_baseline	-2.729e+00	2.956e+00	-0.923	0.363
ARI:mts_baseline	2.120e-01	5.071e-01	0.418	0.678

References

1. Dennis M, Francis DJ, Cirino PT, Schachar R, Barnes MA, Fletcher JM. Why IQ is not a covariate in cognitive studies of neurodevelopmental disorders. Journal of the International Neuropsychological Society. 2009;15(3):331-43.
2. Bates D, Mächler M, Bolker B, Walker S. Fitting linear mixed-effects models using lme4. arXiv preprint arXiv:14065823. 2014.
3. Team MC, Blanchard G, Dickhaus T, Hack N, Konietschke F, Rohmeyer K, et al. Package ‘mutoss’. Statistics in Medicine. 2017;9:811-8.
